# Ectopic expression of *Arabidopsis FD* and *FD PARALOGUE* in rice results in dwarfism with size reduction of spikelets

**DOI:** 10.1038/srep44477

**Published:** 2017-03-14

**Authors:** Seonghoe Jang, Hsing-Yi Li, Mei-Lin Kuo

**Affiliations:** 1 Biotechnology Center in Southern Taiwan (BCST), No. 59 Siraya Blvd., Xinshi Dist., Tainan 74145/Agricultural Biotechnology Research Center, Academia Sinica, No. 128, Sec. 2, Academia Road, Nankang, Taipei 11529, Taiwan; 2 Institute of Tropical Plant Science, National Cheng Kung University, No. 1 University Road, East Dist., Tainan 70101, Taiwan

## Abstract

Key flowering genes, *FD* and *FD PARALOGUE (FDP*) encoding bZIP transcription factors that interact with a FLOWERING LOCUS T (FT) in *Arabidopsis* were ectopically expressed in rice since we found AtFD and AtFDP also interact with HEADING DATE 3a (Hd3a) and RICE FLOWERING LOCUS T 1 (RFT1). Transgenic rice plants overexpressing *AtFD* and *AtFDP* caused reduction in plant height and spikelet size with decreased expression of genes involved in cell elongation without significant flowering time alteration in spite of increased expression of *OsMADS14* and *OsMADS15*, rice homologues of *APETALA1 (AP1*) in the leaves. Simultaneous overexpression of *AtFD* and *AtFDP* enhanced phenotypes seen with overexpression of either single gene while transgenic rice plants expressing *AtFD* or *AtFDP* under the control of phloem-specific *Hd3a* promoter were indistinguishable from wild-type rice. Candidate genes responsible for the phenotypes were identified by comparison of microarray hybridization and their expression pattern was also examined in WT and transgenic rice plants. It has so far not been reported that *AtFD* and *AtFDP* affect cell elongation in plants, and our findings provide novel insight into the possible roles of *AtFD* and *AtFDP* in the mesophyll cells of plants, and potential genetic tools for manipulation of crop architecture.

Formation of a hexameric florigen activation complex (FAC) composed of a rice florigen Hd3a, GF14 proteins (14-3-3 s) and phosphorylated transcription factor OsFD1 has been reported to activate *OsMADS15*, a homologue of *Arabidopsis APETALA1 (AP1*) resulting in flowering^1^. Since interactions between certain isoforms of 14-3-3 and *Arabidopsis* FT have also been shown^2^,^3^ and AtFD is phosphorylated by calcium-dependent protein kinases^4^, without direct evidence it has been assumed that AtFT-AtFD interaction in *Arabidopsis* occurs in a manner similar to rice Hd3a-OsFD1. Recently, OsFD2, a homologue of OsFD1 was proposed to control leaf development through interaction with the same components of a FAC^5^, and this supports the notion that distinct transcription factors may confer the formation of various FAC-like complexes to control programs in plant growth and development.

Hd3a and RFT1 are two florigen genes in rice. The expression of both genes is controlled by a complicated genetic network integrating photoperiodic signaling and circadian clock information^6^. Hd3a is a leaf-borne florigenic signal moving to the shoot apex to induce flowering^7^. Among 13 homologues of *Hd3a* in the rice genome^8^, *RICE FLOWERING LOCUS T 1 (RFT1*) and *FT-like 1 (FT*-*L1*) share extensive sequence similarity with Hd3a and induce early flowering when overexpressed^9^,^10^ indicating that they have the features of floral activators. *RFT1* is also expressed in the leaf vascular tissues, and simultaneous knockdown of *Hd3a* and *RFT1* by RNAi approaches prevents flowering for at least 300 days under inductive short days^11^. Besides promoting flowering, FT homologues are known to play various roles in plant developmental processes although it remains unknown whether formation of FAC-like complexes is necessarily required for their function. Reported roles include controlling tuberization in potato^12^, growth cessation and bud set in *Populus*^13^, leaf shape and inflorescence architecture in tomato^14^, stomatal opening in *Arabidopsis*^15^ and bulb formation in onion^16^.

AtFD, an *Arabidopsis* bZIP transcription factor is preferentially expressed in the shoot apex and required for AtFT to promote flowering through the formation of a complex between them in the shoot apex. Phosphorylation of AtFD at the 282th threonine residue is essential for AtFT-AtFD complex formation and functional complementation of *fd-1*^17^. In the case of OsFD1, phosphorylation of the 192th serine residue has been proposed to be critical for the formation of FAC and activation of *OsMADS15* in the protoplasts^1^. Overexpression of *OsFD1* for the phosphomimetic form (OsFD1 S192E) has been reported to promote flowering although there were no significant flowering time alterations in overexpressors or dsRNAi lines for *OsFD1*^1^. Remarkably, *OsFD* genes are pretty strongly expressed in the leaves and other vegetative organs distinct from the expression pattern of *AtFD* in *Arabidopsis*^5^.

14-3-3s are being found to play key roles in an expanding variety of plant health, growth and developmental processes. Genetic verification of the roles of GF14 proteins as florigen receptors is complicated by gene redundancy and by many processes in which these molecules have been involved^18^. Eight isoforms of GF14 are available in rice and six of them are expressed in the shoot apex^19^. Simultaneous reduction of the expression of four *GF14*s only caused mild reduction of target gene transcription of the FAC indicating that other isoforms might play a compensatory role^1^. Additionally, *GF14c* knock-out mutant rice is seedling-lethal and transgenic rice with *GF14c* overexpression show delayed flowering^19^.

Even though it is still unknown whether all interactions between FT homologues and bZIP proteins are dependent on 14-3-3s, published data demonstrated that a FAC-like complex containing OsFD2 possesses the same components required for the formation of FAC, but is implicated in rice leaf development.

In this work, we observed distinct molecular characteristics in two homologous proteins, AtFT and Hd3a: in binding to bZIP proteins and flowering promotion in rice. We also found that transgenic rice overexpressing *AtFD* and *AtFDP* exhibited dwarfism with reduced organ size without significant alteration of flowering time. Transgenic *Arabidopsis* overexpressing *AtFD* also showed reduced body size as well as early flowering. Both AtFD and AtFDP are able to interact with two rice florigenic proteins, Hd3a and RFT1 in yeast and rice protoplast systems. Transgenic dwarf rice caused by *AtFD*/*AtFDP* overexpression showed reduced expression of genes involved in cell elongation but still responded to gibberellin (GA_3_). Candidate rice genes affected by *AtFD* and *AtFDP* which are likely responsible for the phenotypes were identified. The majority of these candidate genes were found to encode various types of kinases. In view of these results we propose that *AtFD* and *AtFDP* likely influence cell elongation or proliferation through GA biosynthesis control and/or various phosphorylation-mediated signal transduction cascades in rice although it is not clear whether the action of AtFD and/or AtFDP is mediated by the FAC-like complex formation or not. Functional evaluation of the candidate genes identified will be the next step for their potential use in manipulation of crop architecture.

## Results

### *Arabidopsis* FD and FDP interact with Hd3a and RFT1

**Figure 1:**
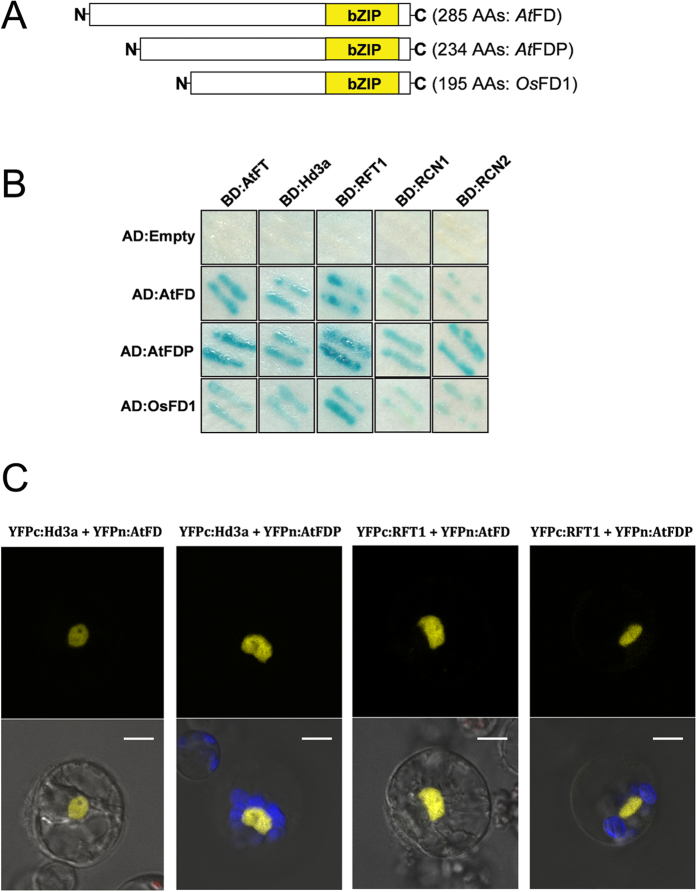
Protein interactions among FTs and FDs. (**A**) Structure of AtFD, AtFDP from *Arabidopsis* and OsFD1 from rice. bZIP domains are marked with yellow boxes. (**B**) AtFD and AtFDP are able to interact with rice phosphatidylethanolamine-binding protein (PEBP) family proteins as well as AtFT in yeast cells. (**C**) Positive interactions in the yeast system between AtFD/AtFDP and rice proteins Hd3a and RFT1 were verified in rice protoplasts by BiFC assays. Scale bars = 5 μm.

To test whether *Arabidopsis* FD and FDP are able to interact with rice Hd3a or RFT1, rice FT homologues, we utilized yeast two-hybrid systems and got positive results for Hd3a, RFT1, AtFD and AtFDP. First, AtFD and AtFDP proteins were co-localized with Hd3a and RFT1 in the nucleus of rice protoplasts derived from mesophyll cells (Supplementary Fig. S1). Although all the bZIP domain proteins, AtFD, AtFDP and rice OsFD1 interacted with phosphatidylethanolamine binding protein (PEBP) family proteins including AtFT, Hd3a, RICE CENTRORADIALIS1 (RCN1) and RCN2 in the yeast systems (Fig. 1), MBP pull-down assays demonstrated that Hd3a does not interact directly with the bZIP proteins such as AtFD, AtFDP and OsFD1. AtFT, however, was able interact with the bZIP proteins in the same system when the MBP:Hd3a was used as a bait protein (Supplementary Fig. S2). This result implies a molecular discrepancy between AtFT and Hd3a in terms of interaction with other proteins including bZIP domain proteins. In the case of Hd3a-OsFD1 interaction, for example, eukaryotic-specific 14-3-3 proteins are required^1^ and in eukaryotic cells including yeast and plant cells those proteins are believed to act as mediators for the Hd3a-OsFD1 interaction. The phenotypes of transgenic rice plants overexpressing *AtFT* and *Hd3a* were also obviously different: rice plants overexpressing *Hd3a* produced panicles from the callus during tissue culture period whereas none of the transgenic rice overexpressing *AtFT* exhibited such extreme early flowering (Supplementary Fig. S3). Results of BiFC *in vivo* assays using rice protoplasts also supported the AtFD/AtFDP-Hd3a and AtFD/AtFDP-RFT1 interactions, although it is unclear whether 14-3-3s are associated for the interactions in the rice cell. Yellow florescence from the reconstructed YFP was observed in the nucleus of rice protoplasts (Fig. 1).

### Overexpression of *AtFD* and *AtFDP* produces sheathed panicles with reduced height in rice

**Figure 2:**
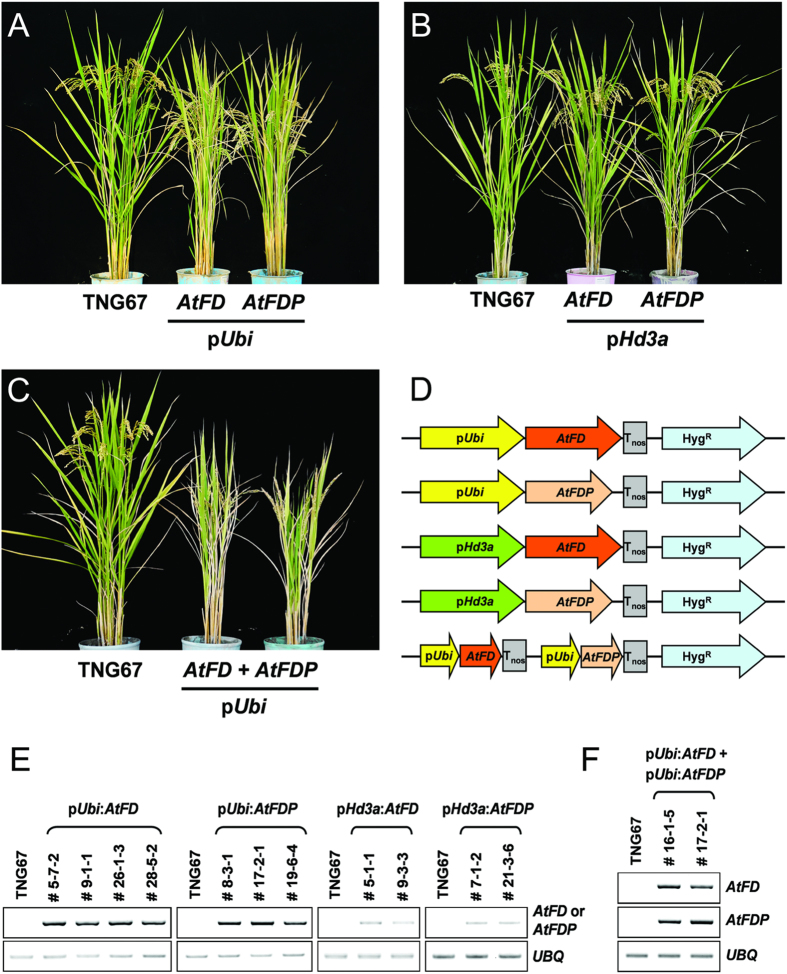
Transgenic rice plants expressing *AtFD* and/or *AtFDP*. (**A**) Transgenic rice plants expressing *AtFD* or *AtFDP* under the control of maize *ubiquitin* promoter show reduced height. (**B**) Phloem-specific expression of *AtFD* or *AtFDP* driven by *Hd3a* promoter does not affect rice architecture. (**C**) Simultaneous overexpression of *AtFD* and *AtFDP* in rice causes more severe dwarfism than either of the single overexpressors. (**D**) Simplified schema of each plasmid used for rice transformation in (**A**,**B**) and (**C**). (**E,F**) RT-PCR analyses for expression of introduced *AtFD* and/or *AtFDP* in the leaves of independent T3 transgenic lines. Original gel images are available in Supplementary Fig. S11A.

**Figure 3:**
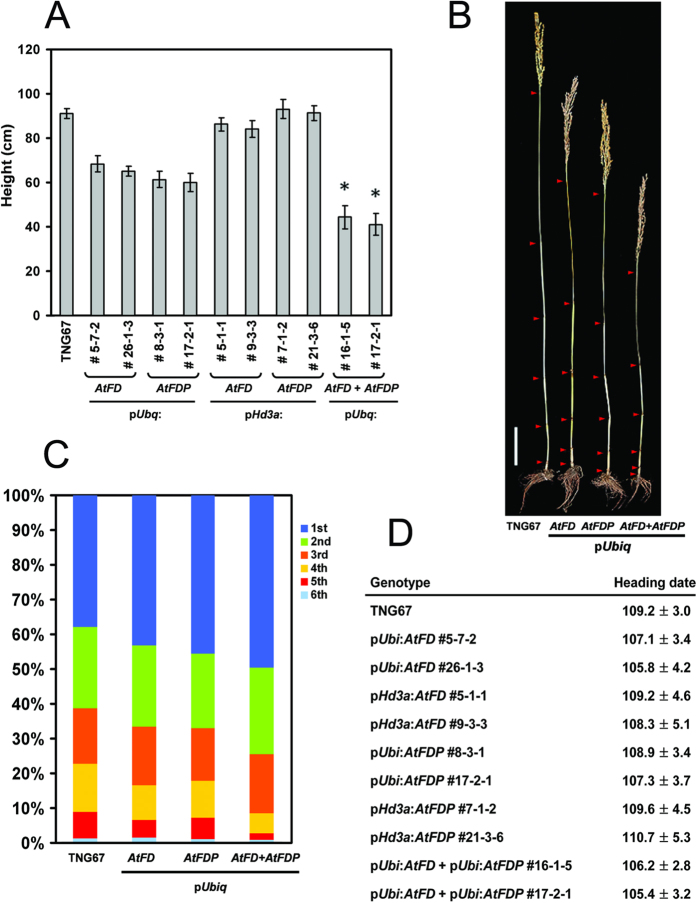
Characterization of transgenic rice plants. (**A**) Measurement of plant height. Main culms of six to twelve plants per line were used for height measurement except panicle length. Values are means ± SD (n = 8–12, *P < 0.0001, Student’s *t* test). (**B**) Reduced plant height in transgenic rice plants. Arrowheads present each node. Scale bar = 10 cm (**C**) Proportion of each internode in transgenic rice plants. A representative plant from each line of p*Ubi:AtFD* (#5-7-2), p*Ubi:AtFDP* (#17-2-1) and p*Ubi:AtFD*-p*Ubi:AtFDP* (#16-1-5) was used together with TNG67. (**D**) Measurement of heading date from eight to twenty individual plants per line. Heading date is recorded based on number of days from sowing to the emergence of the first panicle. Values are means ± SD (n = 8–12).

**Figure 4:**
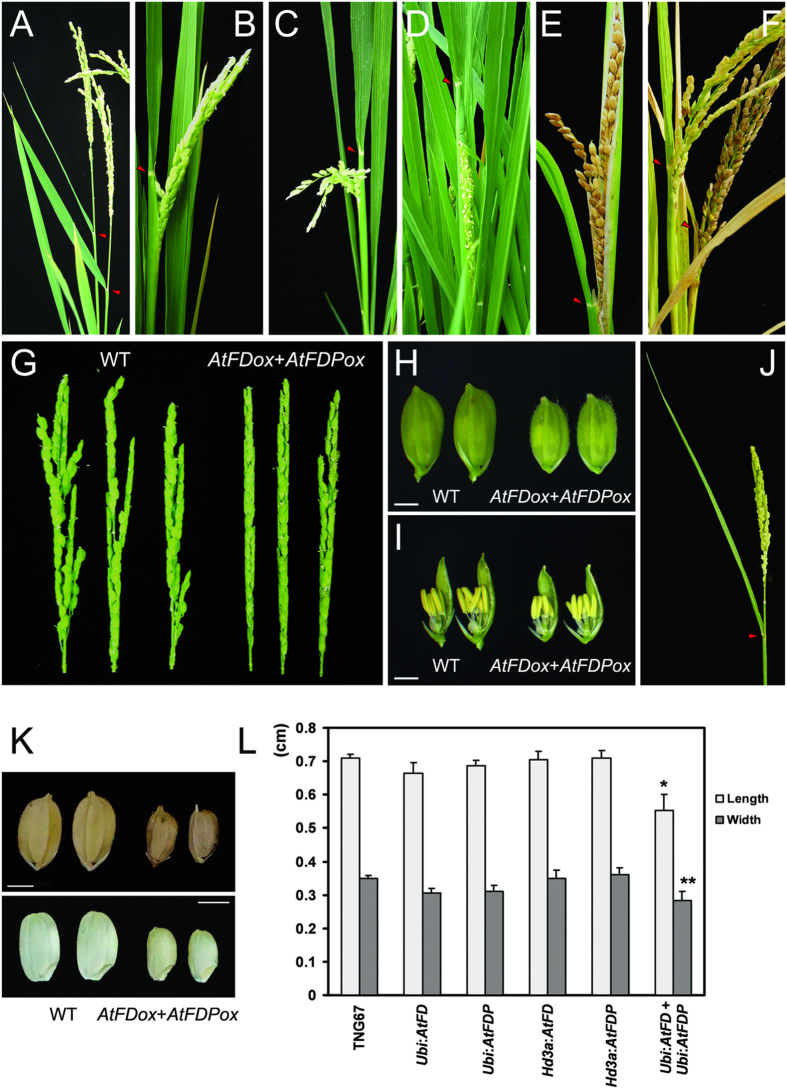
Transgenic rice plants exhibit sheathed panicles, small spikelets and grains. (**A**) Panicles of wild-type TNG67. (**B,D,E**) Panicles of *AtFD* overexpressors. Sheathed panicles were observed and sometimes grains became mature in the sheath (**E**). (**C**) A panicle of a *AtFDP* overexpressor. (**F**) Panicles of *AtFD*-*AtFDP* dual overexpressor showing sheathed panicles with reduced fertility. The first three panicles from each independent homozygous transgenic and control plants were carefully checked for the sheathed panicle phenotype. All the panicles examined were sheathed in all *AtFD*, *AtFDP* and *AtFD*-*AtFDP* dual overexpressing transgenic rice plants; this was not observed in TNG67 or transgenic rice containing the empty vector. (**G**) Panicles of TNG67 (left three panicles) and *AtFD*-*AtFDP* dual overexpressors. Each spikelet of transgenic panicles is smaller than WT and panicles are also more compact (**G,H,I**). (**H,I**) Transgenic rice plants for *AtFD*-*AtFDP* dual overexpression produce smaller spikelets containing tiny floral organs (right two spikelets). (**J**) A panicle of transgenic rice overexpressing *OsbZIP23*/*OsbZIP72* is indistinguishable from WT. (**K,L**) Grain size is also reduced in *AtFD*-*AtFDP* dual overexpressors. More than 20 grains randomly picked up from each genotype were used for size measurement and similar results were obtained from T3 and T4 generation plants. Values are means ± SD (n > 20, *P < 0.005; **P < 0.05, Student’s *t* test).

**Figure 5:**
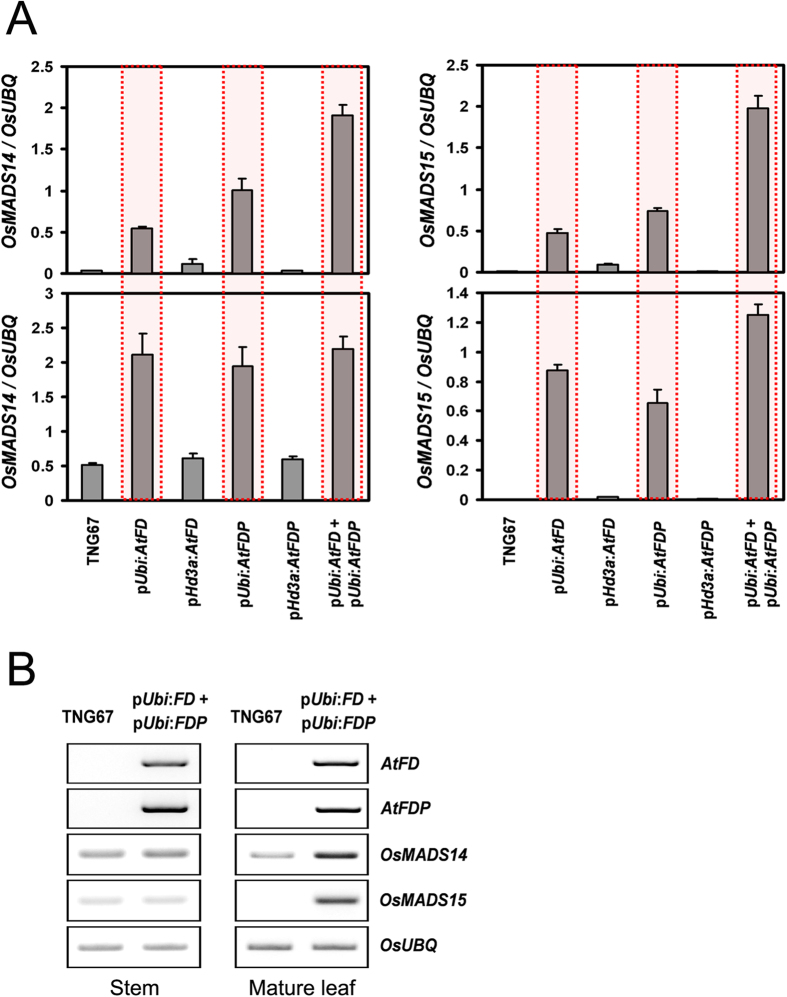
Differential expression of two rice *AP1* homologues, *OsMADS14* and *OsMADS15* between WT and transgenic rice plants. (**A**) Expression of *OsMADS14* and *OsMADS15* is up-regulated only in p*Ubi:AtFD*, p*Ubi:AtFDP* and p*Ubi:AtFD*-p*Ubi:AtFDP* double plants: in the aboveground parts of 50-day old plants (top) and in mature leaves of 100-day old plants. Values are means ± SD of three biological replicates. (**B**) Expression of *OsMADS14* and *OsMADS15* is increased in the mature leaves (right panel) but not in the stems of transgenic rice plants at the heading stage. Original gel images are available in Supplementary Fig. S11B.

Since AtFD and AtFDP interact with Hd3a and RFT1, the effect of AtFD and AtFDP on rice development was investigated by transgenic approaches. Expression level of each transgene was investigated by RT-PCR using total RNA extracts from leaves of each transgenic rice plant. Compared with the wild-type control, TNG67, transgenic rice plants containing p*Ubi:AtFD* or p*Ubi:AtFDP* construct showed reduced plant height and grain size without significant alteration in heading date (Figs 2,3 and 4). Of particular note, sheathed panicles resulted in reduced fertility and consequently caused loss of yield. Measuring node numbers and the length of internodes in each transgenic plant revealed that the elongation of lower internodes was severely retarded (Fig. 3). Smaller size spikelets and grains were also observed in the transgenic rice plants indicating that overexpression of *AtFD* and/or *AtFDP* may affect cell elongation during rice development (Fig. 4). Notably, transcripts of *OsMADS14* and *OsMADS15* were accumulated in the leaves but not in the stems of transgenic plants although no significant changes in heading date were observed (Fig. 5).

### Simultaneous overexpression of *AtFD* and *AtFDP* enhanced the phenotypes observed in p*Ubi:AtFD* or p*Ubi:AtFDP* transgenic rice plants

To investigate whether *AtFD* and *AtFDP* act in an additive manner in rice, a genetic construct for simultaneous expression of the two genes, *AtFD* and *AtFDP* was generated and introduced into rice plants through *Agrobacterium*-mediated transformation methods. The insertion of the construct into the rice genome was confirmed by genomic PCR (Supplementary Fig. S4) and the simultaneous expression of *AtFD* and *AtFDP* in the leaves of each transgenic plant was also verified by RT-PCR (Fig. 2). The transgenic plants exhibited dwarfism with severe reduction in grain size; lower internodes, in particular, showed heavily retarded elongation (Fig. 3) and the size of epidermal cells in the stem was also reduced (Supplementary Fig. S5). However, the transgenic plants responded to gibberellin (GA_3_) in cell elongation (Supplementary Fig. S6). The size of spikelets and the epidermal cells of floral organs such as palea were smaller than those of control wild-type plants as well (Supplementary Fig. S5). The length and width of grains from the transgenic rice plants were decreased by an average of 22.5% and 19%, respectively (Fig. 4). All the panicles were sheathed and fertility was also severely affected; only a few grains were properly matured in each plant (Fig. 4). Compared with wild-type pollen, the viability as well as the size of pollen from transgenic rice plants was decreased (Supplementary Fig. S5). Remarkably, the expression of *OsMADS14* and *OsMADS15*, rice *AP1* homologues was up-regulated only in the leaves but not in the stems of *AtFD*-*AtFDP* dual overexpressors controlled by ubiquitin promoter suggesting a distinct effect of *AtFD* and/or *AtFDP* on different cell types (Fig. 5). Since mesophyll cells are abundant in the leaves but not in the stems of rice^20^ and also phloem-specific expression of *AtFD* and/or *AtFDP* did not show any obvious phenotypes such as dwarfism or the reduced spikelet size shown in *AtFD*/*AtFDP* overexpressors driven by *ubiquitin* promoter, the effect of *AtFD*/*AtFDP* responsible for those phenotypes seems to mainly occur in mesophyll cells. Otherwise, leaf-preferentially expressed *Hd3a*/*RFT1* may be responsible for the induction of *OsMADS14* and *OsMADS15* only in the leaves of *AtFD*/*AtFDP* overexpressors^7^,^11^.

### Phloem-specific expression of *AtFD* and *AtFDP* by the *Hd3a* promoter in rice

*AtFD* and *AtFDP* were specifically expressed in the phloem tissue of rice by using *Hd3a* promoter. Expression of the transgenes was checked by RT-PCR using total RNAs extracted from the leaves of each transgenic plant. Unlike transgenic rice plants carrying p*Ubi:AtFD* and p*Ubi:AtFDP*, transgenic rice plants containing p*Hd3a:AtFD* or p*Hd3a:AtFDP* did not show any significant phenotypic alteration in plant height and grain size (Figs 2 and 3). If AtFD and/or AtFDP interact with Hd3a (or RFT1) in rice phloem companion cells, alteration of heading date can be expected by the up-regulation of downstream genes such as *OsMADS15* (early heading) or retardation of Hd3a movement to the shoot apex (delayed heading). However, no significant changes in either heading date or expression of *OsMADS14* and *OsMADS15* were detected in the transgenic plants (Figs 3 and 5). To verify the activity of *Hd3a* promoter we used, p*Hd3a:OsFD1* transgenic rice plants were generated. Interestingly, they showed an early heading time with slightly reduced plant height but had no significant reduction in spikelet/grain size demonstrating a molecular functional discrepancy between *AtFD*/*AtFDP* and *OsFD1* in rice (Supplementary Fig. S7).

### Identification of candidate genes responsible for the reduction in stem elongation and spikelet size

**Figure 6:**
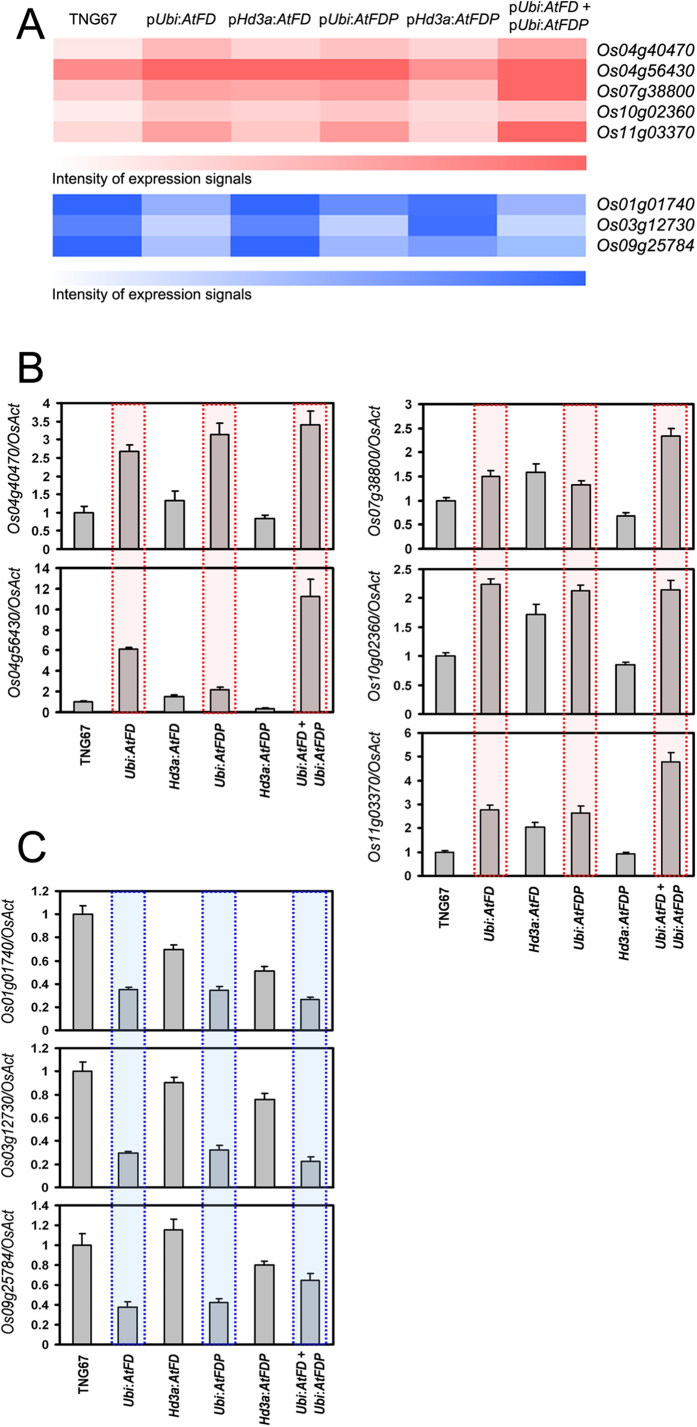
Confirmation of the expression patterns of candidate genes identified by microarray-based expression changes in the transgenic plants that showed phenotypes. (**A**) A heat map showing expression level of candidate genes in each genotype through microarray hybridization. Red color and blue color represent up- and down-regulated candidate genes, respectively. Expression of candidates identified by microarray analyses as up-regulated genes (**B**) and down-regulated genes (**C**) in 50-day old plants of p*Ubi:AtFD*, p*Ubi:AtFDP* and p*Ubi:AtFD*-p*Ubi:AtFDP* double plants. Values are means ± SD of three PCR replicates.

Since transgenic rice plants of p*Ubi:AtFD*, p*Ubi:AtFDP* and p*Ubi:AtFD*-p*Ubi:AtFDP* showed a significant growth defect especially in stem elongation and spikelet development compared to the wild type, TNG67 or transgenic rice plants expressing *AtFD* or *AtFDP* in a phloem-specific manner, candidate rice genes responsible for the defective phenotypes of *AtFD*, *AtFDP* and *AtFD*-*AtFDP* dual overexpressors were identified by analyses of microarray hybridization experiments. Total RNAs from the above-ground parts of 50-day-old transgenic plants for p*Ubi:AtFD*, p*Ubi:AtFDP*, p*Hd3a:AtFD*, p*Hd3a:AtFDP* and p*Ubi:AtFD*-p*Ubi:AtFDP* together with control TNG67 were extracted for microarray hybridization. By comparison of each microarray hybridization result, we identified eight genes displaying significant changes of expression in plants that showed phenotypes: Os04g40470 (encoding a cytochrome p450 family protein), Os04g56430 (encoding a cysteine-rich receptor-like protein kinase), Os07g38800 (encoding a lectin-like receptor kinase), Os10g02360 (encoding a wall-associated kinase), Os11g03370 (encoding a NAC-domain containing transcription factor), Os01g01740 (encoding a protein kinase-domain protein), Os03g12730 (encoding a leucine rich repeat-receptor like kinase) and Os09g25784 (encoding a auxin-induced protein 5NG4). First, we selected up-regulated genes in p*Ubi:AtFD*, p*Ubi:AtFDP* and *AtFD*-*AtFDP* dual overexpressors without significant changes of expression in p*Hd3a:AtFD* and p*Hd3a:AtFDP* compared with WT. Next, among the candidates, we narrowed down genes showing higher levels of expression in *AtFD*-*AtFDP* dual overexpressors, which showed more severe phenotypes than either of the single gene overexpressors. Down-regulated genes were also selected by the same logic. Thus, the first five genes, Os04g40470, Os04g56430, Os07g38800, Os10g02360 and Os11g03370 were picked up as up-regulated genes and the other three genes, Os01g01740, Os03g12730 and Os09g25784 were selected as genes down-regulated by *AtFD* and *AtFDP* overexpression with phenotypes. Quantitative RT-PCR analyses showed that some genes are similar but others such as Os07g38800, Os10g02360 and Os09g25784 have different expression patterns compared to the results of microarray hybridization (Fig. 6).

### Expression changes of candidate genes responsible for short stems

**Figure 7:**
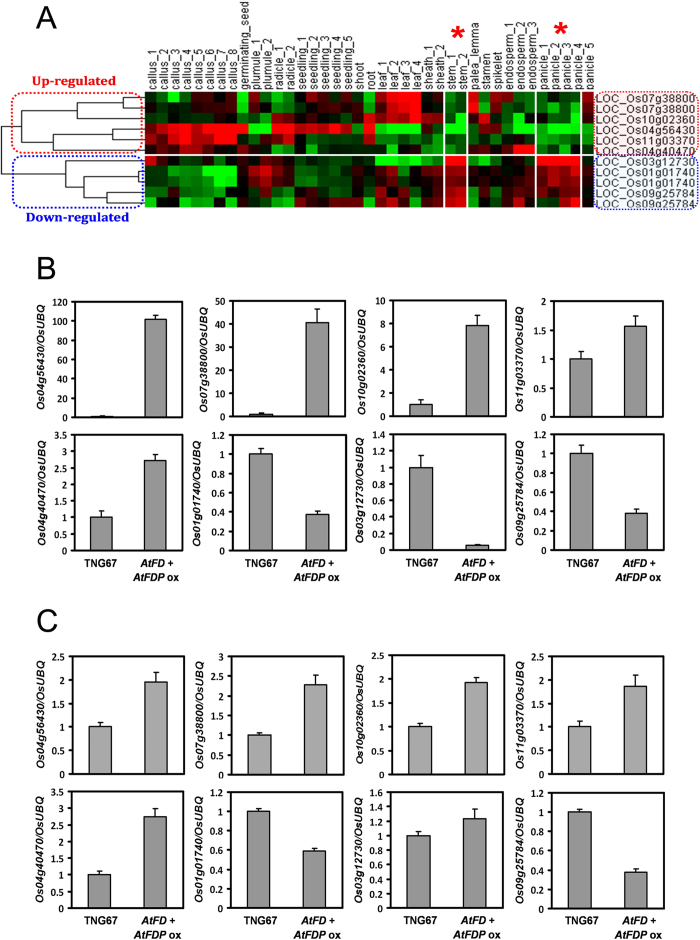
Generation of a heat map showing spatiotemporal expression and expression analyses of candidate genes in the stems and panicles. (**A**) Up-regulated and down-regulated genes are differentially grouped into two clades. Expression of up-regulated genes in stems and panicles is low and that of down-regulated genes is high, in common. Red asterisks indicate stems and panicles. In the heat map, the red color represents an increase and the green color represents a decrease in gene expression, respectively. Verification of expression level of candidate genes in stems (**B**) and young panicles (**C**) between WT and *AtFD*-*AtFDP* dual overexpressors. Stem and panicle samples we used for expression analyses are matched with the stem_2 stage (heading stage) and panicle_4 stage (developing panicle with length between 40 and 50 mm), respectively in the heat map in (**A**). Values are means ± SD of three PCR replicates.

We investigated the expression patterns of each gene identified using different rice organs at different developmental stages (Supplementary Fig. S8). The spatiotemporal expression patterns of the candidate genes are similar to the microarray results available in the public database (http://www.ricearray.org). Notably, candidate genes up-regulated by *AtFD* and *AtFDP* overexpression exhibited lower expression levels, whereas genes down-regulated by *AtFD* and *AtFDP* overexpression showed a higher expression level in stems and panicles of wild-type rice, in common, based on the public microarray database (Fig. 7). To test whether this expressional relationship is correct, we investigated expression of candidate genes in panicles and stems of *AtFD*-*AtFDP* dual overexpressors compared with WT. Indeed, all candidate genes exhibited similar changes in expression between WT and *AtFD*-*AtFDP* dual overexpressing stems and panicles (except for Os03g12730 expression in panicles) compared with those in the above-ground parts of 50-day-old WT and *AtFD*-*AtFDP* dual overexpressors (Figs 6 and 7).

**Figure 8:**
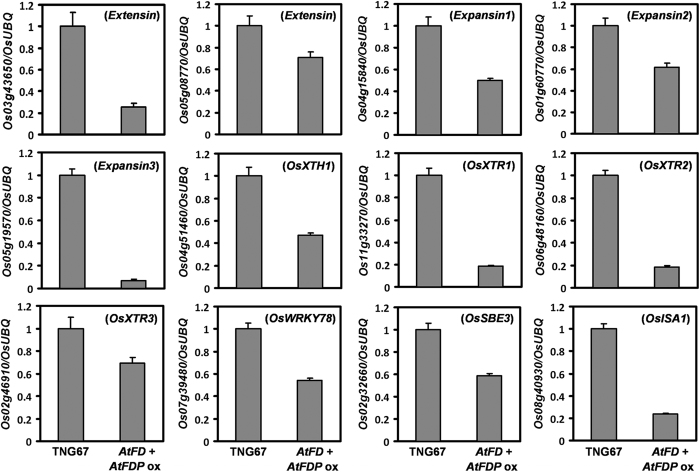
Expression analyses of genes involved in cell elongation in the stems between 100-day-old WT and *AtFD*-*AtFDP* dual expressing plants. Values are means ± SD of three PCR replicates.

Since we observed short stems composed of smaller epidermal cells in the transgenic rice plants (Supplementary Fig. S5), we compared expression levels of several genes involved in cell elongation in the stems of *AtFD*-*AtFDP* dual overexpressors and WT rice plants. Reduced level of expression from all the genes examined was detected in the transgenic stems indicating retarded cell elongation (Fig. 8).

## Discussion

As florigen-interacting proteins, bZIP transcription factors AtFD and AtFDP are regarded as important players in the flowering of *Arabidopsis*^21^. Rice OsFD1 as a component of FAC of rice has also been shown to be responsible for the activation of downstream genes such as *OsMADS15*, a floral meristem identity gene^1^. However, it seems that transgenic rice plants with overexpression or knockdown of *OsFD1* do not show significant alterations in heading date despite overexpression of *OsFD1* for the phosphomimetic form (OsFD1 S192E) causes early heading^1^. Based on the results of yeast two-hybrid system and BiFC assays in rice protoplasts, AtFD and AtFDP are able to interact not only with AtFT but also Hd3a and RFT1. In rice, 14-3-3 proteins are intracellular Hd3a receptors and the Hd3a-OsFD1 interaction is dependent on the existence of 14-3-3 proteins, which are eukaryote-specific in glutathione S-transferase (GST) pull-down assays^1^,^22^. It has also been suggested that the interaction of AtFT with AtFD is indirect, mediated by the 14-3-3 adaptors, with yeast 14-3-3s being able to substitute for plant 14-3-3s in yeast two-hybrid assays for the formation of FAC in the nucleus^1^ although no direct evidence is available^4^,^17^. Interestingly, however, we observed distinct interaction profiles between Hd3a and AtFT with different bZIP proteins in the MBP pull-down assays. We observed positive interactions between AtFT and bZIP proteins under the same conditions as for the negative interaction between Hd3a and OsFD1, which mirrors published results^1^ implying molecular discrepancy between the two proteins, AtFT and Hd3a. Moreover, transgenic rice plants overexpressing *Hd3a* always produce spikelets/panicles from the callus during transformation, a trait which was not observed in transgenic rice with *AtFT* overexpression.

Transgenic rice overexpressing *AtFD* and *AtFDP* simultaneously exhibited reduced plant height and size of organs including spikelets, and these phenotypes were more severe than either of the single gene overexpressors. However, phloem-specific expression of *AtFD* or *AtFDP* did not cause these phenotypic alterations suggesting that the two bZIP proteins had roles in mesophyll cells. Indeed, transgenic *Arabidopsis* expressing *AtFD* under the control of mesophyll cell-specific *CHLOROPHYLL A/B BINDING* PROTEIN 3 (*CAB3*) promoter exhibit reduced plant body size with dwarfism^23^ (Supplementary Fig. S9). Remarkably, overexpressors of *OsbZIP23* and *OsbZIP72* encoding proteins interacting with Hd3a, RFT1 and GF14c were indistinguishable from wild-type rice implying that the phenotypes driven by *AtFD* and *AtFDP* overexpression are gene-specific (Fig. 4, Supplementary Fig. S10). A recent report showing an early heading of transgenic rice plants (TNG67 cultivar) containing p*Ubi:PaFD*, an *AtFD* homologue of *Phalaenopsis* orchid also support this notion^24^.

Of note, the reduced spikelet size of p*Ubi:OsFD1* or p*Ubi:OsFD1*(S192E) plants indicates that smaller spikelet size is not linked to heading date because there is a significant difference in heading date between p*Ubi:OsFD1* and p*Ubi:OsFD1*(S192E) transgenic rice plants although the effect of *OsFD1* on rice height is unknown^1^. If the action of AtFD and/or AtFDP introduced into rice is through the formation of FAC-like complexes, it would be worthwhile checking whether GF14s are available in phloem companion cells since no phenotypic alterations were observed in transgenic plants expressing *AtFD* or *AtFDP* in the phloem companion cells. Even though there were no significant changes in heading date, notably, the expression of *OsMADS14* and *OsMADS15* was increased in the leaves but not in the stems of p*Ubi:AtFD*, p*Ubi:AtFDP* and p*Ubi:AtFD*-p*Ubi:AtFDP* double transgenic rice plants indicating that downstream genes affected by *AtFD* and/or *AtFDP* are likely to be distinct based on cell types. Also, retarded stem elongation may influence the heading date of the transgenic plants. In that case, the transition of the shoot apex to the reproductive phase may occur early based on increased expression of two rice *AP1* genes but retarded elongation of stem may compromise the early flowering phenotype since days for panicle emergence are counted as heading date in rice. Moreover, we cannot exclude the possibility that formation of complexes may occur between introduced AtFD/AtFDP and endogenous RCN proteins whose transcripts are preferentially abundant in the vegetative and reproductive meristems. Actually, *RCN1*/*RCN2*-overexpressing transgenic rice plants under the control of *35S* promoter resulted in delayed flowering^25^.

*AtFD* has been reported to be a flowering activator encoding a bZIP transcription factor that interacts with AtFT, with *fd* mutants exhibiting delayed flowering^17^,^26^. However, there has been no report about its possible effect on vegetative tissue or organ development. In *Arabidopsis*, Abe *et al*.^17^ demonstrated that p*35S:AtFD* rescued the late flowering of *fd-1*. Although they did not describe the reduced plant size, the complemented mutant was very tiny. We observed such a small sized plants caused by p*35S:AtFD* even in wild-type *Arabidopsis* with a mild early flowering phenotype although phloem-specific *AtFD* expression in *fd*-3 mutant did not exhibit such phenotypes showing similar plant size and flowering time to control plants (Supplementary Fig. S9). These results gained from transgenic *Arabidopsis* approaches are similar to the phenotypes we observed in our transgenic rice plants suggesting common effects of *AtFD* or *AtFDP* in mesophyll cells of the two model plant species. In addition, expression of *OsFD1* under the control of shoot apex-specific *AtFD* promoter was able to rescue the late flowering phenotype of *fd-3* without affecting plant size indicating that *OsFD1* can functionally replace the *AtFD* gene in *Arabidopsis* (Supplementary Fig. S9).

Fukazawa *et al*.^27^,^28^ reported that tobacco bZIP-domain protein, REPRESSION OF SHOOT GROWTH (RSG) is also implicated in cell elongation linked to active GA biosynthesis. In addition, RSG interacts with 14-3-3 proteins^29^, which is similar to several bZIP proteins including wheat EmBP1^30^, *Arabidopsis* FD, FDP, rice OsFDs^5^, OsbZIP23 and OsbZIP72 (Supplementary Fig. S10). BZI-1, another tobacco bZIP protein has been implicated in the fine-tuning of auxin-mediated transcription in plants^31^. Although several rice bZIP proteins interact with Hd3a or RFT1, it is difficult to anticipate whether all of them play similar roles in plant development (Supplementary Fig. S10). For example, overexpression of *OsbZIP72*, which is regarded as a positive regulator of ABA response and drought tolerance^32^, did not cause any dwarfism or reduction in organ size in rice despite its binding pattern being similar to those seen in AtFD, AtFDP and OsFD1 with florigenic proteins and GF14c in yeast systems (Supplementary Fig. S10, Fig. 4). Since bZIP proteins are also known to form homo and/or heterodimers^33^, ectopic expression of bZIP proteins may induce novel phenotypes by interrupting original interactions and/or formation of *de novo* interactions resulting in transcriptional changes in the cells. Recruitment of transcriptional co-activators and co-repressors can also not be excluded in the formation of FT-FD complexes for the downstream gene regulation^2^. Moreover, distinct FAC-like complexes depending on transcription factors and/or PEBPs may occur to regulate various developmental and physiological processes in plants. OsFD2, a homologue of OsFD1 can be an example of the latter since OsFD2 forms a FAC with Hd3a and 14-3-3 for controlling leaf development^5^.

Overexpression of *AtFD* and *AtFDP* in rice caused dwarfism with reduced size of the plant body, which is likely due to retarded cell elongation. Indeed, expression of a series of genes involved in cell elongation such as extensins^34^ and expansins^35^ is reduced in the stems of *AtFD*-*AtFDP* dual overexpressors. In addition, genes that encode cell-wall loosening enzymes necessary for cell elongation such as xyloglucan endotransglycosylase-related (XTR) proteins^36^,^37^, xyloglucan endotransglycosylase/hydrolase (XTH) proteins^38^ as well as *OsWRKY78* that encode a transcription factor regulating stem elongation and its putative targets, *OsSBE3* and *ISA1* show reduced expression in the short stems of *AtFD*-*AtFDP* overexpressors compared to WT indicating defective cell elongation in the dual gene overexpressors^39^. Reduced expression level of genes encoding expansins, extensins and XTHs is also observed in p*35S:AtFD* transgenic *Arabidopsis* compared to WT (Supplementary Fig. S9) demonstrating a common molecular effect of *AtFD* in rice and *Arabidopsis*. Since the transgenic dual overexpressors still respond to exogenous GA_3_ treatment, phenotypes of dwarfism and small organs are likely linked to impaired GA biosynthesis in plants.

Candidate rice genes responsible for the phenotypes were identified based on transcriptomic analyses using microarrays. Interestingly, the majority of the candidates were found to encode various types of protein kinases whose expression is influenced by overexpression of *AtFD* and *AtFDP* in rice. It is also known that bZIP proteins such as AtFD and RSG are phosphorylated by calcium-dependent protein kinases in *Arabidopsis*^4^ and tobacco^40^.

The dynamic nature of the plant cell wall allows growing cells to expand. Although multiple receptor-like kinases (RLKs) that likely play a role in regulating cell wall function have been identified, many questions remain unanswered about the downstream signaling targets and effects on plant growth and development^41^. Recently, it was reported that rice leucine-rich repeat receptor-like kinase 1 (LRK1) overexpression increased rice growth by promoting cellular proliferation. One of our candidate genes, Os03g12730 encoding a similar type kinase to LRK1 showed reduced expression in transgenic dwarf plants^42^. Moreover, higher expression of Os11g03370 that codes for a NAC transcription factor is observed in transgenic rice containing p*Ubi:AtFD*/*AtFDP* and, recently, it was reported that transgenic rice plants constitutively expressing *OsNAC2* (Os04g38720) caused shorter plant height with shorter spikelets, which may support our results since both NAC transcription factors, Os11g03370 and OsNAC2 belong to the same NAM/CUC3 subgroup of NAC transcription factor family in rice^43^,^44^. Functional characterization of the candidate genes identified in this study as being likely responsible for the phenotypes of transgenic rice may provide valuable information for potential application in the manipulation of plant architecture in cereals.

## Methods

### Plant materials and growth conditions

Japonica rice (*Oryza sativa* L.) variety Tainung67 (TNG67) and *Arabidopsis* Columbia-0 (Col) were used as wild type. TNG67, a photoperiod insensitive flowering rice cultivar containing defective *Hd1* and *Ehd1* sequences the same as those of Taichung65 (T65)^45^,^46^ was used to produce transgenic rice plants and the transgenic plants were grown in a growth chamber (14 h L, 28 °C/10 h D, 26 °C) for 2 weeks after germination and moved to the outdoor GMO greenhouse of the Academia Sinica Biotechnology Center in Southern Taiwan (23°04′N 120°17′E). Phenotypes of transgenic rice were examined from the second crop period (July to November) of 2011 to the first crop period (February to June) 2016. Transgenic plants used for analyses in this work are all T3 or T4 independent homozygous lines. Control rice plants with an empty vector were also generated and it was verified that they are almost identical to TNG67 in heading date, plant height and grain size. Generally, *Arabidopsis* plants were grown in the growth chamber under LD conditions (16/8-h photoperiod at 100 μmol m^−2^ s^−1^) at 22 °C. *fd-3* in the Columbia background has been previously described^17^. For *Arabidopsis* flowering time measurement, 8 to 12 plants per line were counted for total leaf numbers when their first flowers were at anthesis. Days of heading of 10 to 12 rice plants per each line were measured when panicles were emerged.

### GA_3_ treatment

Rice seedlings were germinated and incubated on MS media for 10 days and transferred to test tubes containing water (mock) or GA_3_ (5 μM) solution. The plants were grown for 2 days under 12 h-light photoperiod at constant temperature of 28 °C, and then taken out for cryo-scanning electron microscopy^47^.

### Yeast two-hybrid experiments and BiFC assays

*AtFT, Hd3a, RCN1 and RCN2 full-length* ORFs were cloned in-frame in the pBD-GAL4 Cam vector (Stratagene) to generate bait constructs. The pAD-GAL4 vector (Stratagene) was used for generation of preys including pAD:AtFD, pAD:AtFDP and pAD:OsFD1 constructs. X-gal filter assays were performed as described previously^24^,^47^. For cellular localization of Hd3a, RFT1, AtFD and AtFDP in rice, either yellow florescence protein (YFP):gateway (GW) or cyan fluorescent protein (CFP):GW vector was used for the florescence fusion as described previously^24^. For bimolecular florescence complementation (BiFC) assays in rice, each cDNA of Hd3a and RFT1 was cloned into pVYCE vector for Hd3a:cYFP and RFT1:cYFP fusions. *AtFD* and *AtFDP* cDNAs were cloned into pVYNE vector for nYFP:AtFD and nYFP:AtFDP fusions, respectively^48^,^49^. Isolation and transfection of rice protoplasts were followed as described by Zhang *et al*.^50^ and images of cells with fluorescence were taken by confocal microscopy (LSM 510 META NLO DuoScan, Carl Zeiss).

### MBP pull-down assay

pMAL-4cx vector (NEB) and pET201 vector^51^ were used for production of maltose binding protein (MBP) fusion proteins and 6× histidine (His)-tag fusion proteins, respectively. *AtFT* and *Hd3a* cDNA fragments for each coding region with XbaI/SalI and EcoRI/SalI ends, respectively, were ligated into SpeI/SalI or EcoRI/SalI-treated pMAL-4cx vector for MBP:AtFT and MBP:Hd3a proteins. *AtFD*, *AtFDP* and *OsFD1* cDNA fragments digested by SpeI/XhoI were introduced between NheI and XhoI sites of pET201 for His-tag fusion. Transformed cells [*E. coli* strain Rosetta (DE3), Novagen] were grown and the tagged proteins were induced with 1 mM IPTG when the culture reached an OD600 value of 0.6. After 20 hours of induction at 16 °C, the cells were harvested and homogenized by sonication in either MBP buffer (20 mM Tris pH 8.0, 1 mM EDTA, 200 mM NaCl), for MBP-tagged proteins, or 1XPBS (137 mM NaCl, 2.7 mM KCl, 10 mM Na_2_HPO_4_, 1.8 mM KH_2_PO_4_), for HIS-tagged proteins. MBP-tagged proteins were immobilized onto Amylose Resin (NEB) and incubated with His-tagged proteins-containing crude extract in the binding buffer (20 mM Tris pH 8.0, 1 mM EDTA, 100 mM NaCl, 0.1% NP40, 1 mM PMSF) for 3 hours at 4 °C with constant rotation. The unbound residues were removed by washing with binding buffer 6 times. Proteins with affinity to the resins were separated on 12% SDS polyacrylamide gels and detected by western blot.

### Expression analysis

Total RNAs from plant materials were extracted using an RNeasy Plant Mini Kit (Qiagen) and treated with RNase-free DNase (Invitrogen) following the manufacturer’s protocol to remove any residual genomic DNA. DNase-treated RNA was subjected to reverse transcriptase reactions using oligo (dT) primer and Superscript III reverse transcriptase (Invitrogen) according to the manufacturer’s protocol. Subsequent PCR was performed with the first-strand cDNA mixture and EX-Taq polymerase (Takara Bio). qPCR was performed on a CFX96TM real-time system (Bio-Rad) using Maxima SYBR Green qPCR Master Mix (Thermo). The primers used for quantification are listed in Supplementary Table S1. For PCR, each sample was analyzed in triplicate. The run protocol was: denaturation at 95 °C for 10 min and annealing/extension repeated 45 times (95 °C for 15 s and 60 °C for 30 s, data acquisition was performed). Housekeeping genes such as *OsUBQ*^11^, *OsAct*^52^, *AtPEX4*^53^ and *AtACT*^54^ were included in the reactions as internal controls for normalizing the variations in the amount of cDNA used^55^. The threshold cycle (C_T_) was automatically determined for each reaction by the system set with default parameters. The specificity of the quantitative reverse transcription-PCR (qRT-PCR) was determined by curve analysis of the amplified products using the standard method installed in the system. Information on primers used is presented in Supplementary Table S1.

### Plant transformation and analyses of transgenic plants

For p*SUC2:AtFD*, p*SUC2:AtFDP* and p*AtFD:OsFD1* constructs, *AtFD*, *AtFDP* and *OsFD1* entry clones were inserted into p*SUC2*:GW and/or p*AtFD*:GW destination vectors, respectively^24^,^56^. For p*CAB3:AtFD*, 1,537 bp *CAB3* promoter was fused to *AtFD* as described previously^57^. All plasmids for plant transformation were introduced into *Agrobacterium* strain GV3101 (pMP90RK) and transformed into Col WT or *fd-3* plants by the floral-dip method^58^. For overexpression of target genes in rice, the binary vector pGA3426 and its derivative pGA3777 were used, and each transgene construct was introduced into the rice genome by *Agrobacterium*-mediated transformation^59^. For p*Hd3a:AtFD*, p*Hd3a:AtFDP* and p*Hd3a:OsFD1*, *ubiquitin* promoter was exchanged for 2.2 Kb-promoter of *Hd3a*; 2,153 bp *Hd3a* promoter fragment amplified by primers 5′CGCGGATCCAGGCATCAGATTAAGACAGCAACGCAG3′ and 5′GCGACTAGTCGATCTTGCAAAAAACCCTGAAGGTTTATAG3′ were used. Restriction enzyme sites for BamHI and SpeI are underlined, respectively, which is longer than the one Hayama *et al*.^60^ used previously and then *AtFD*, *AtFDP* and *OsFD1* were introduced in the plasmid, respectively. More than 15 independent primary transgenic plants were obtained and analyzed, and at least two independent homozygous lines were selected for phenotypic description.

### Microarray analyses

The whole above ground parts of two independent homozygous plants per each genotype (50-day old plants) were harvested for RNA extraction for microarray. We used the Rice Whole Genome OneArray v1.1 (Phalanx Biotech Group, Taiwan), which contains 22,003 DNA oligonucleotide probes, and each probe is a 60-mer designed in the sense direction. Among the probes, 21,179 probes corresponded to the annotated genes in the RGAP v.6.1 and BGI database. In addition, we included 824 control probes. The detailed descriptions of the gene array list are available from http://www.phalanx.com.tw/products/RiOA_Probe.php. Fluorescent antisense RNA (aRNA) targets were prepared from 1 μg total RNA samples with the OneArray Amino Allyl aRNA Amplification Kit (Phalanx Biotech Group, Taiwan) and Cy5 dyes (Amersham Pharmacia, Piscataway, NJ, USA). Fluorescent targets were hybridized to the Rice OneArray with Phalanx hybridization buffer using Phalanx Hybridization System. After hybridization for 16 h at 50 °C, non-specific binding targets were washed away by three different washing steps (Wash I, 42 °C 5 min; Wash II, 42 °C, 5 min; 25 °C, 5 min; Wash III, rinse 20 times), and the slides were dried by centrifugation and scanned by an Agilent G2505C scanner (Agilent Technologies, Santa Clara, CA, USA). The Cy5 fluorescent intensities of each spot were analyzed by GenePix 4.1 software (Molecular Devices). The signal intensity of each spot was loaded into Rosetta Resolver System (Rosetta Bio-software) to process data analysis. The error model of the Rosetta Resolver System could remove both systematic and random errors from the data. We filtered out the spots whose flag was less than 0. Spots that passed the criteria were normalized by 50% media scaling normalization method. The technical repeat data was tested by Pearson correlation coefficient calculation to check the reproducibility (R value > 0.975). Normalized spot intensities were transformed to gene expression log2 ratios between the control and treatment groups. The interest spots which show significant differences were selected by log2 ratio ≥1 or log2 ratio ≤−1 and *P* < 0.05. Two independent biological replicates of hybridizations were performed.

### Hierarchical cluster analysis

The raw data of expression values were downloaded from the microarray experiment, and the developmental transcriptomes of rice were dissected using the Internet website “Rice Oligonucleotide Array Database” (http://www.ricearray.org). Then, the raw data were inputted into Gene Cluster 3.0 (http://bonsai.hgc.jp/~mdehoon/software/cluster/). The graphic of clustering heat map was created using with Alok Saldanha’s Java TreeView v1.1.6r2.

## Additional Information

**Accession codes:** AtFD (At4g35900), AtFDP (At2g17770, AtbZIP27), Hd3a (Os06g06320), OsFD1 (Os09g36910), RCN1 (Os11g05470), RCN2 (Os02g32950), RFT1 (Os06g06300), GEO accession number for microarray results (GSE93822)

**How to cite this article:** Jang, S. *et al*. Ectopic expression of *Arabidopsis* *FD* and *FD PARALOGUE* in rice results in dwarfism with size reduction of spikelets. *Sci. Rep.* **7**, 44477; doi: 10.1038/srep44477 (2017).

**Publisher's note:** Springer Nature remains neutral with regard to jurisdictional claims in published maps and institutional affiliations.

## Supplementary Material

Supplementary Information

## References

[b1] Taoka K.-i. . 14-3-3 proteins act as intracellular receptors for rice Hd3a florigen. Nature 476, 332–335, doi: http://www.nature.com/nature/journal/v476/n7360/abs/nature10272.html#supplementary-information (2011). 21804566 10.1038/nature10272

[b2] Ho W. W. H. & Weigel D. The Plant Cell 26, 552–564, doi: 10.1105/tpc.113.115220 (2014). 24532592 PMC3967025

[b3] Niwa M. . The Plant Cell 25, 1228–1242, doi: 10.1105/tpc.112.109090 (2013). 23613197 PMC3663264

[b4] Kawamoto N., Sasabe M., Endo M., Machida Y. & Araki T. Scientific Reports 5, 8341, doi: 10.1038/srep08341 (2015). 25661797 PMC4321167

[b5] Tsuji H., Nakamura H., Taoka K.-i. & Shimamoto K. Plant and Cell Physiology 54, 385–397, doi: 10.1093/pcp/pct005 (2013). 23324168 PMC3589828

[b6] Tsuji H., Taoka K.-i. & Shimamoto K. Current Opinion in Plant Biology 14, 45–52, doi: 10.1016/j.pbi.2010.08.016 (2011). 20864385

[b7] Tamaki S., Matsuo S., Wong H. L., Yokoi S. & Shimamoto K. Science 316, 1033–1036, doi: 10.1126/science.1141753 (2007). 17446351

[b8] Faure S., Higgins J., Turner A. & Laurie D. A. Genetics 176, 599–609, doi: 10.1534/genetics.106.069500 (2007). 17339225 PMC1893030

[b9] Izawa T. . Genes & Development 16, 2006–2020, doi: 10.1101/gad.999202 (2002). 12154129 PMC186415

[b10] Kojima S. . Plant and Cell Physiology 43, 1096–1105, doi: 10.1093/pcp/pcf156 (2002). 12407188

[b11] Komiya R., Ikegami A., Tamaki S., Yokoi S. & Shimamoto K. Development 135, 767–774 (2008). 18223202 10.1242/dev.008631

[b12] Navarro C. . Nature 478, 119–122, doi: http://www.nature.com/nature/journal/v478/n7367/abs/nature10431.html#supplementary-information (2011). 21947007 10.1038/nature10431

[b13] Hsu C.-Y. . Proceedings of the National Academy of Sciences of the United States of America 108, 10756–10761, doi: 10.1073/pnas.1104713108 (2011). PMC3127867 21653885

[b14] Krieger U., Lippman Z. B. & Zamir D. Nat Genet 42, 459–463, doi: http://www.nature.com/ng/journal/v42/n5/suppinfo/ng.550_S1.html (2010). 20348958 10.1038/ng.550

[b15] Kinoshita T. . Current Biology 21, 1232–1238, doi: 10.1016/j.cub.2011.06.025 (2011). 21737277

[b16] Lee R., Baldwin S., Kenel F., McCallum J. & Macknight R. Nat Commun 4, doi: 10.1038/ncomms3884 (2013). 24300952

[b17] Abe M. . Science 309, 1052–1056, doi: 10.1126/science.1115983 (2005). 16099979

[b18] Denison F. C., Paul A.-L., Zupanska A. K. & Ferl R. J. Seminars in Cell & Developmental Biology 22, 720–727, doi: 10.1016/j.semcdb.2011.08.006 (2011). 21907297

[b19] Purwestri Y. A., Ogaki Y., Tamaki S., Tsuji H. & Shimamoto K. Plant and Cell Physiology 50, 429–438, doi: 10.1093/pcp/pcp012 (2009). 19179350

[b20] Maiti R., Satya P., Rajkumar D. & Ramaswamy A. Crop plant anatomy. (Wallingford, UK, CAB International, 2012)

[b21] Jaeger K. E., Pullen N., Lamzin S., Morris R. J. & Wigge P. A. The Plant Cell 25, 820–833, doi: 10.1105/tpc.113.109355 (2013). 23543784 PMC3634691

[b22] Lozano-Durán R. & Robatzek S. Molecular Plant-Microbe Interactions 28, 511–518, doi: 10.1094/MPMI-10-14-0322-CR (2015). 25584723

[b23] Susek R. E., Ausubel F. M. & Chory J. Cell 74,787–799. 7690685 10.1016/0092-8674(93)90459-4

[b24] Jang S., Choi S.-C., Li H.-Y., An G. & Schmelzer E. PLoS ONE 10, e0134987, doi: 10.1371/journal.pone.0134987 (2015). 26317412 PMC4552788

[b25] Nakagawa M., Shimamoto K. & Kyozuka J. The Plant Journal 29, 743–750, doi: 10.1046/j.1365-313X.2002.01255.x (2002). 12148532

[b26] Wigge P. A. . Science 309, 1056–1059 (2005). 16099980 10.1126/science.1114358

[b27] Fukazawa J. . Plant Signaling & Behavior 6, 26–28, doi: 10.4161/psb.6.1.14114 (2011). 21248488 PMC3122000

[b28] Fukazawa J. . The Plant Cell 12, 901–916 (2000). 10852936 10.1105/tpc.12.6.901 PMC149092

[b29] Ishida S., Fukazawa J., Yuasa T. & Takahashi Y. The Plant Cell 16, 2641–2651, doi: 10.1105/tpc.104.024604 (2004). 15377759 PMC520961

[b30] Schultz T. F., Medina J., Hill A. & Quatrano R. S. The Plant Cell 10, 837–847 (1998). 9596641 10.1105/tpc.10.5.837 PMC144375

[b31] Heinekamp T. . The Plant Journal 38, 298–309, doi: 10.1111/j.1365-313X.2004.02043.x (2004). 15078332

[b32] Lu G., Gao C., Zheng X. & Han B. Planta 229, 605–615, doi: 10.1007/s00425-008-0857-3 (2009). 19048288

[b33] Iven T. . The Plant Journal 63, 155–166, doi: 10.1111/j.1365-313X.2010.04230.x (2010). 20409000

[b34] Lamport D. T. A., Kieliszewski M. J., Chen Y. & Cannon M. C. Plant Physiology 156, 11–19, doi: 10.1104/pp.110.169011 (2011). 21415277 PMC3091064

[b35] Cho H. T. & Kende H. The Plant Cell 9, 1661–1671, doi: 10.1105/tpc.9.9.1661 (1997). 9338967 PMC157041

[b36] Duan K. . The Plant Journal 47, 519–531, doi: 10.1111/j.1365-313X.2006.02804.x (2006). 16827922

[b37] Uozu S., Tanaka-Ueguchi M., Kitano H., Hattori K. & Matsuoka M. Plant Physiology 122, 853–860 (2000). 10712549 10.1104/pp.122.3.853 PMC58921

[b38] Yokoyama R., Rose J. K. C. & Nishitani K. Classification and Expression Analysis. Plant Physiology 134, 1088–1099, doi: 10.1104/pp.103.035261 (2004). 14988479 PMC389933

[b39] Zhang C.-Q. . Planta 234, 541–554, doi: 10.1007/s00425-011-1423-y (2011). 21547461

[b40] Ishida S., Yuasa T., Nakata M. & Takahashi Y. The Plant Cell 20, 3273–3288, doi: 10.1105/tpc.107.057489 (2008). 19106376 PMC2630431

[b41] Steinwand B. J. & Kieber J. J. Plant Physiology 153, 479–484, doi: 10.1104/pp.110.155887 (2010). 20410434 PMC2879783

[b42] Zha X. . Plant Biotechnology Journal 7, 611–620, doi: 10.1111/j.1467-7652.2009.00428.x (2009). 19619185

[b43] Chen X. . The Plant Journal 82, 302–314, doi: 10.1111/tpj.12819 (2015). 25754802

[b44] Nuruzzaman M. . Genome-wide analysis of NAC transcription factor family in rice. Gene. 465, 30–44, doi: 10.1016/j.gene.2010.06.008 (2010). 20600702

[b45] Doi K. . Genes Dev. 18, 926–936 (2004) 15078816 10.1101/gad.1189604 PMC395851

[b46] Wang J. D. . Botanical Studies 54, 12, doi: 10.1186/1999-3110-54-12 (2013) PMC5432754 28510861

[b47] Jang S. Plant and Cell Physiology 56, 2234–2247, doi: 10.1093/pcp/pcv130 (2015). 26493518

[b48] Citovsky V. . Journal of Molecular Biology 362, 1120–1131, doi: 10.1016/j.jmb.2006.08.017 (2006). 16949607

[b49] Tzfira T. . Plant Molecular Biology 57, 503–516, doi: 10.1007/s11103-005-0340-5 (2005). 15821977

[b50] Zhang Y. . Plant Methods 7, 30–30, doi: 10.1186/1746-4811-7-30 (2011). 21961694 PMC3203094

[b51] Hajheidari M., Farrona S., Huettel B., Koncz Z. & Koncz C. CDKF;1 and CDKD Protein Kinases Regulate Phosphorylation of Serine Residues in the C-Terminal Domain of Arabidopsis RNA Polymerase II. The Plant Cell 24, 1626–1642, doi: 10.1105/tpc.112.096834 (2012). 22547781 PMC3398568

[b52] Song W. . Annals of Botany, doi: 10.1093/aob/mct212 (2013).

[b53] Gregis V. . Genome Biology 14, 1–26, doi: 10.1186/gb-2013-14-6-r56 (2013).

[b54] Jang S., Torti S. & Coupland G. Plant J 60 (2009). 10.1111/j.1365-313X.2009.03986.x 19656342

[b55] Guénin S. . Journal of Experimental Botany 60, 487–493, doi: 10.1093/jxb/ern305 (2009). 19264760

[b56] An H. . Development 131, 3615–3626, doi: 10.1242/dev.01231 (2004). 15229176

[b57] Ranjan A., Fiene G., Fackendahl P. & Hoecker U. Development 138, 1851–1862, doi: 10.1242/dev.061036 (2011). 21447551

[b58] Clough S. J. & Bent A. F. The Plant Journal 16, 735–743, doi: 10.1046/j.1365-313x.1998.00343.x (1998). 10069079

[b59] Kim S.-R., Lee D.-Y., Yang J.-I., Moon S. & An G. Journal of Plant Biology 52, 73–78, doi: 10.1007/s12374-008-9008-4 (2009).

[b60] Hayama R., Yokoi S., Tamaki S., Yano M. & Shimamoto K. Nature 422, 719–722, doi: http://www.nature.com/nature/journal/v422/n6933/suppinfo/nature01549_S1.html (2003). 12700762 10.1038/nature01549

