# Supplementary material for: Ectopic expression of Arabidopsis FD and FD PARALOGUE in rice results in dwarfism with size reduction of spikelets
Source: Sci Rep. 2017 Mar 14;7:44477. doi: 10.1038/srep44477 (PMC5349553; doi:10.1038/srep44477)
Supplement: Supplementary Information [file srep44477-s1.pdf]

Supplementary information (11 figures and 1 table)

Ectopic expression of *Arabidopsis FD* and *FD PARALOGUE* in rice results in dwarfism with size reduction of spikelets

Seonghoe Jang[1, 2, *], Hsing-Yi Li[1], Mei-Lin Kuo[1]

[1]Biotechnology Center in Southern Taiwan (BCST), No. 59 Siraya Blvd., Xinshi Dist., Tainan 74145/Agricultural Biotechnology Research Center, Academia Sinica, No. 128, Sec. 2, Academia Road, Nankang, Taipei 11529, Taiwan.

[2]Institute of Tropical Plant Science, National Cheng Kung University, No. 1 University Road, East Dist., Tainan 70101, Taiwan

Legends for supplementary figures

Figure S1. Co-localization of two rice florigenic proteins, Hd3a and RFT1 with *Arabidopsis* bZIP proteins, AtFD and AtFDP in the nucleus of rice protoplasts. Co-localization of AtFD (A) and AtFDP (B) with Hd3a and RFT1 is shown. Bar = 10 μm

Figure S2. MBP pull-down assays. MBP:*At*FT (left) and MBP:Hd3a were pulled down by amylose resin and prey proteins were detected by anti-*His* antibody. Interactions between AtFT and bZIP proteins including AtFD, AtFDP and OsFD1 are shown by MBP pull-down assays. Input represents 1.3% of the amount used for pull-down. Original blot images with protein molecular-weight markers (kDa) are at the bottom.

Figure S3. Transgenic rice plants overexpressing *Hd3a* (top) and *Arabidopsis FT*. More than 15 independent individual plants were examined for each construct.

Figure S4. Verification of an intact construct for dual gene overexpression in plants by genomic PCR. WT: genomic DNAs isolated from a wild-type rice plant were used

as PCR templates. P: plasmid DNAs (positive control) were used as PCR templates. PCR results from five independent T1 plants are shown together with a negative (WT) and positive (P) controls. Binding sites of primers are marked with arrowheads.

Figure S5. Epidermal cells of stem, palea and pollens of WT (A, C, E) and *AtFD-AtFDP* dual overexpressor (B, D, F). The percentage of viable pollen was calculated relative to the total pollen counted (E, F). Bar = 100 μm (E, F). To examine the pollen grains, five mature flowers were collected 2 days prior to anthesis and fixed in 70% (v/v) ethanol at room temperature until use. Anthers from mature flowers were dissected and the pollen grains were stained with I2–KI staining (0.2% iodine and 2% potassium iodide). Pollen grains that stained black were judged as viable and those that stained yellow or light red were considered sterile. The pollen grains were observed under a bright field microscope (DM4000B, Leica, Germany).

Figure S6. Transgenic rice for *AtFD-AtFDP* dual overexpression still responds to a phytohormone, GA3. (A) Application of GA3 (5 μM) for 2 days induced stem elongation in *AtFD-AtFDP* dual overexpressor as well as WT. The red arrowheads indicate nodes in the culm of rice. (B) Epidermal cells of culm in WT (top) and *AtFD-AtFDP* dual overexpressor with (right) or without GA3 treatment. Bar = 50 μm.

Figure S7. Transgenic rice plants harboring p*Hd3a*:*OsFD1* show an early heading time with slightly reduced plant height. (A) Transgenic rice expressing *OsFD1* under the control of *Hd3a* promoter flower on average up to 16 days earlier than control plants (in the case of TNG67 vs. #2-2-4) and their height is also reduced. Values are means±SD (n > 12, * $P < 0.001$; ** $P < 0.005$, Student's *t* test). Grain size of p*Hd3a*:*OsFD1* plants is similar to that of WT (mm; n > 30). (B) *OsFD1* expression in the mature leaves (80-day old stage) is examined by RT-PCR with the following primers: (*OsFD1*-RTF) 5'GCGATGGAGGACGACGAG3' and (*OsFD1*-RTR) 5'GAATGGCGCGGAGAGCAC3' The expression level of *OsFD1* in the transgenic rice is higher than that of a control plant (TNG67). The numbers in parentheses indicate the numbers of PCR cycles. Expression of *OsMADS14* and *OsMADS15* is also significantly increased in the leaves of transgenic plants.

Figure S8. Spatiotemporal expression patterns of genes identified as potentially being responsible for the phenotypes, dwarfism and small organs through reduced cell elongation or proliferation in transgenic rice plants. Values are means±SD of three biological replicates. R, root; L, leaf; LJ, laminar joint region; LS, leaf sheath; S, culm (50 days old) or stem (110 days old); FL, flag leaf; P, panicle.

Figure S9. Transgenic *Arabidopsis* for p*35S*:*AtFD* in Col background (top) and p*SUC2*:*AtFD* in *fd-3* background. (A) The plant size of p*35S*:*AtFD* is smaller than the WT and leaves are wrinkled and rolled. Transgenic *Arabidopsis* for p*35S*:*AtFD* shows mild early flowering under long days (LDs, 16 h L). Flowering time was measured by counting the total number of leaves at flowering under LDs. RL and CL mean rosette leaf and cauline leaf, respectively. Error bars indicate SD (n=10). (B) Expression of genes involved in cell elongation in p*35S*:*AtFD* and WT plants (Expansins, Extensins and Xyloglucan endotransglucosylase/hydrolase genes; Supplementary Table S1). Rosette leaves of 20-day old plants were harvested for RNA extraction. Values are means±SD of three PCR replicates. (C) The *SUC2* promoter is expressed specifically in the companion cells of the phloem and not in the meristem or in young leaf primordia[61]. (D) Expression of *AtFD* under the control of mesophyll cell-specific *CAB3* promoter[25,57] causes small body size, dwarfism with slightly early flowering in *Arabidopsis*. Flowering time was measured by counting the total number of leaves at flowering under LDs. RL and CL mean rosette leaf and cauline leaf, respectively. (E) Complementation of *fd-3* with p*AtFD*:*OsFD1*. Two independent homozygous lines for p*AtFD*:*OsFD1 fd-3* were examined for flowering phenotypes under long days. Flowering time was measured by counting the total number of leaves at flowering

under LDs. RL and CL mean rosette leaf and cauline leaf, respectively. LN means leaf number. Error bars indicate SD (n=8-12).

61. Imlau, A., Truernit, E. & Sauer, N. *The Plant Cell* 11, 309-322 (1999).

Figure S10. Protein-to-protein interactions of rice bZIP proteins. (A) Rice bZIP proteins that interact with rice florigenic proteins, Hd3a or RFT1 (OsbZIP23, Os02g52780; OsbZIP24, Os02g58670; OsbZIP46, Os06g10880; OsbZIP69, Os08g43600; OsbZIP72, Os09g28310)[62]. (B) Carboxyl-terminal region containing bZIP domain of OsbZIP72 is important in interaction with Hd3a and RFT1, which is similar to OsFD1. (C) Differential interaction profile between GF14c, a 14-3-3 protein and rice bZIP proteins identified as binding proteins of Hd3a or RFT1.

62. Nijhawan, A., Jain, M., Tyagi, A. K. & Khurana, J. P. *Plant Physiology* **146**, 333-350, doi:10.1104/pp.107.112821 (2008).

Figure S11. Original gel images used for main figures. Gel photos used for Figure 2E, 2F (A) and Figure 5B (B).

Table S1. List of primers used in this research.

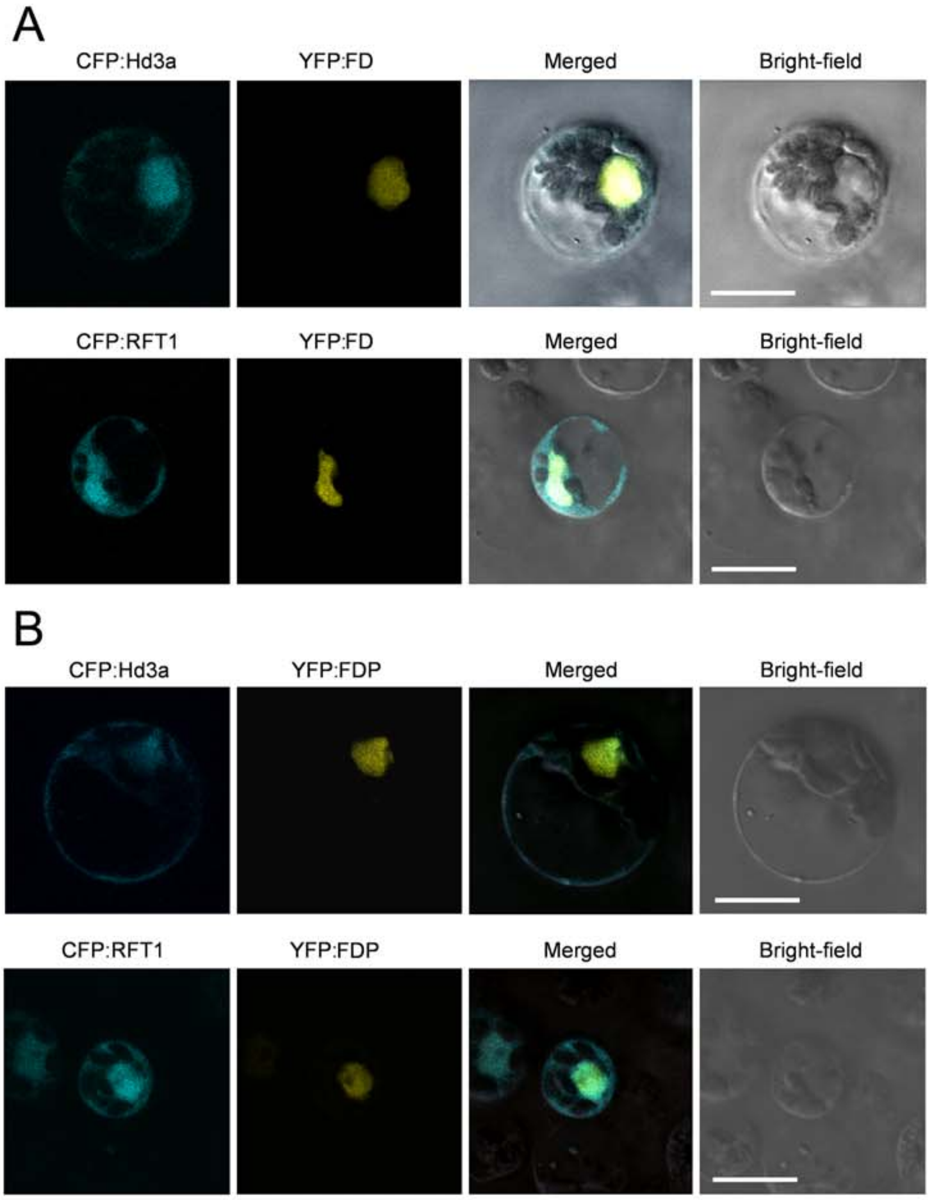

Supplementary figure 1

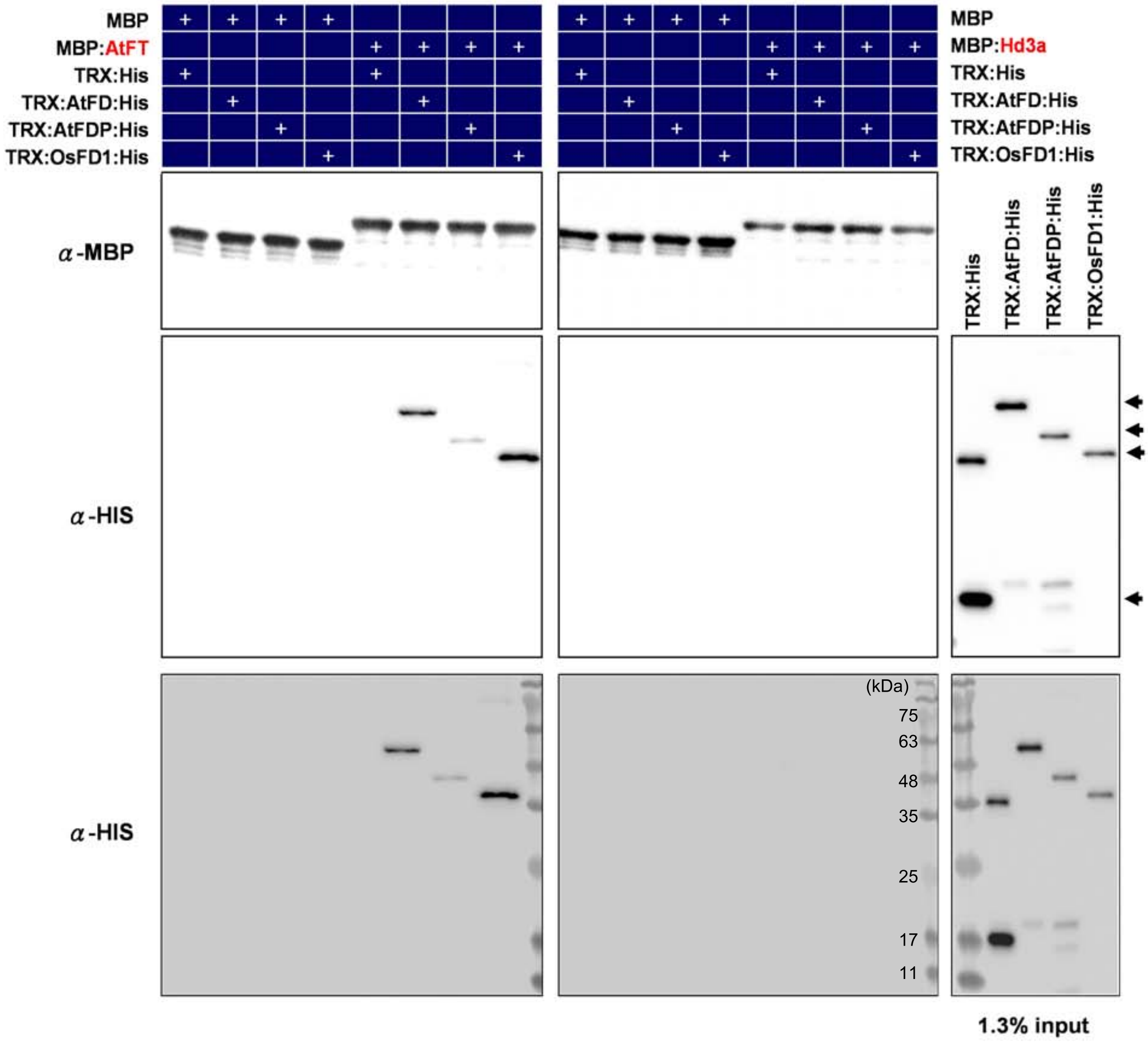

Supplementary figure 2

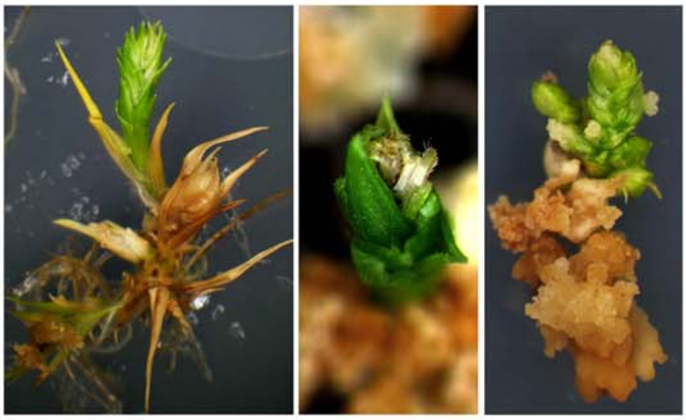

p*Ubi*:*Hd3a*

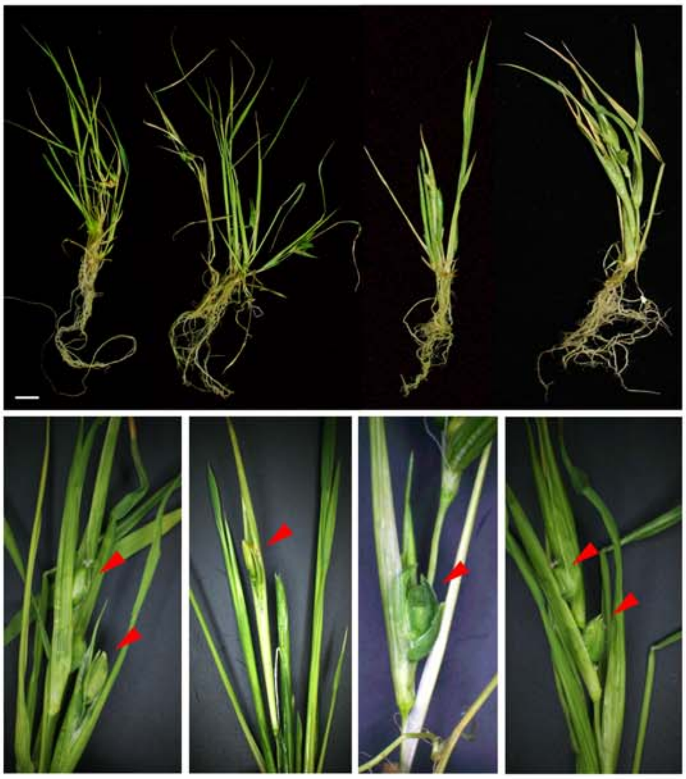

p*Ubi*:*AtFT*

Supplementary figure 3

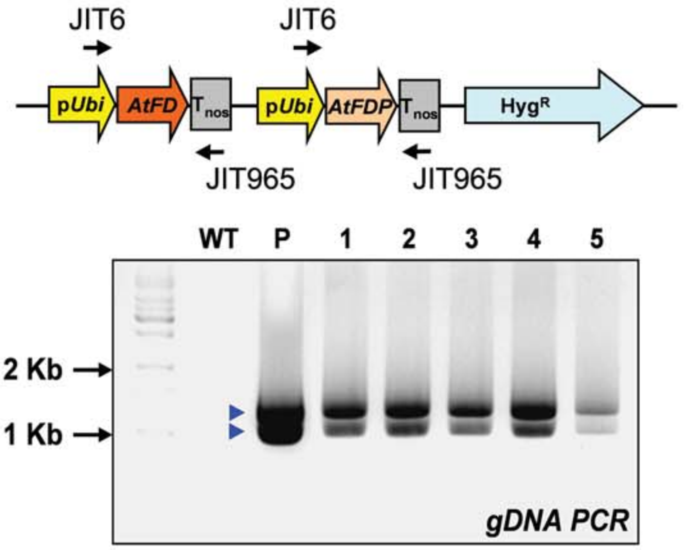

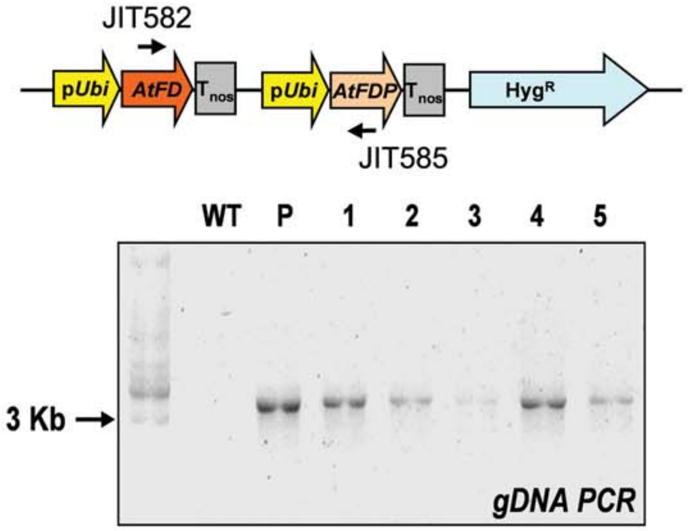

JIT6: 5’ TTGTCGATGCTCACCCTG 3’

JIT965: 5’ AGACCGGCAACAGGATTCAATC 3’

JIT582: 5’ CTAAACCGACACAGCCCTCATC 3'

JIT585: 5' GACAACCAAACTTTGCAGATT 3'

Supplementary figure 4

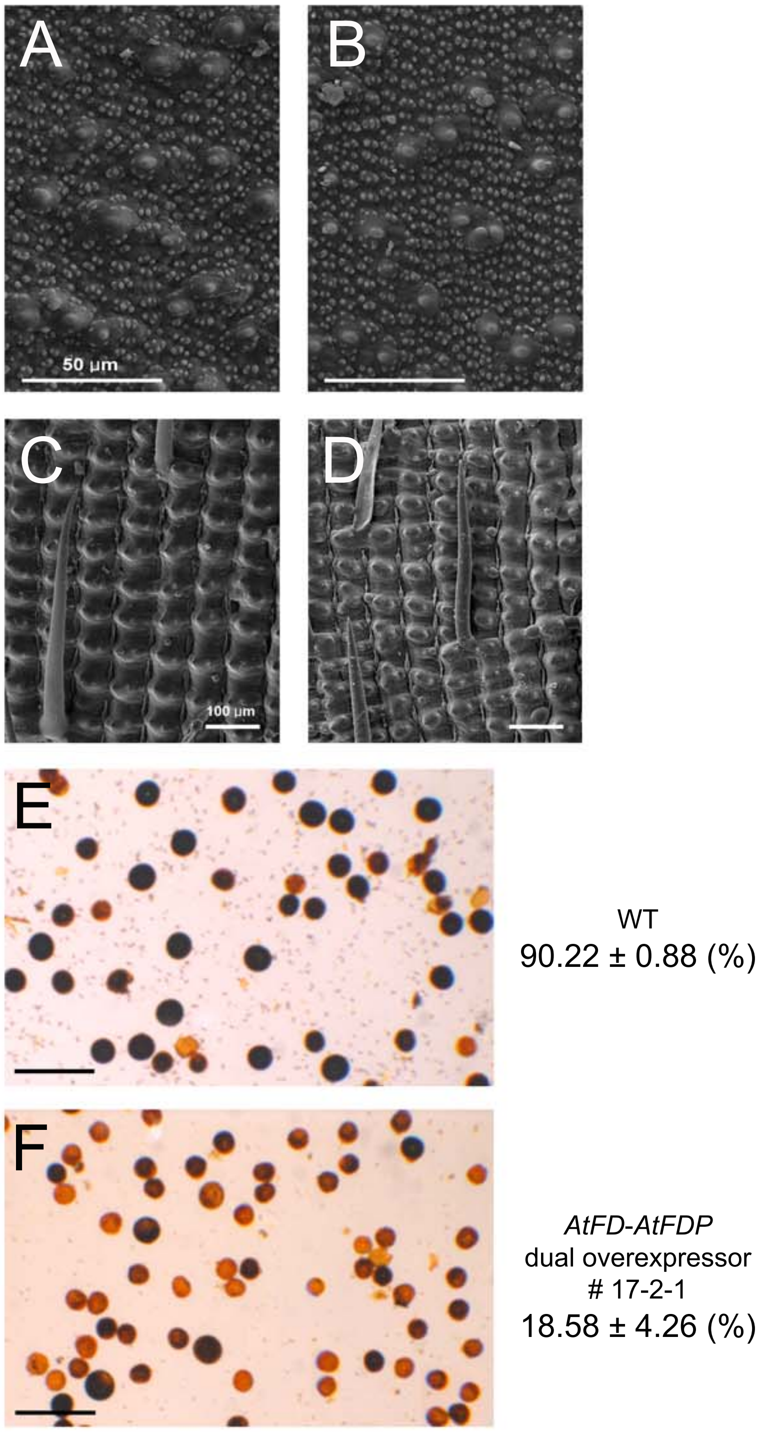

WT
90.22 ± 0.88 (%)

AtFD-AtFDP
dual overexpressor
17-2-1
18.58 ± 4.26 (%)

Supplementary figure 5

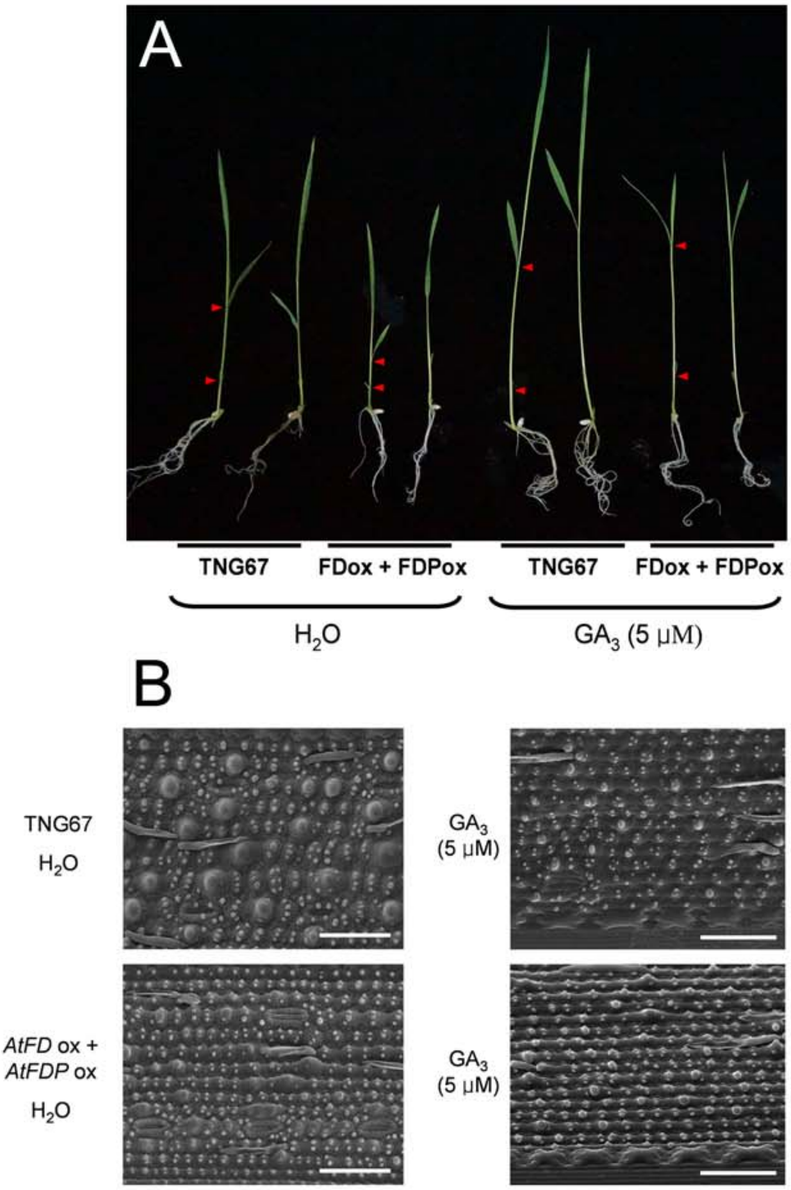

Supplementary figure 6

A

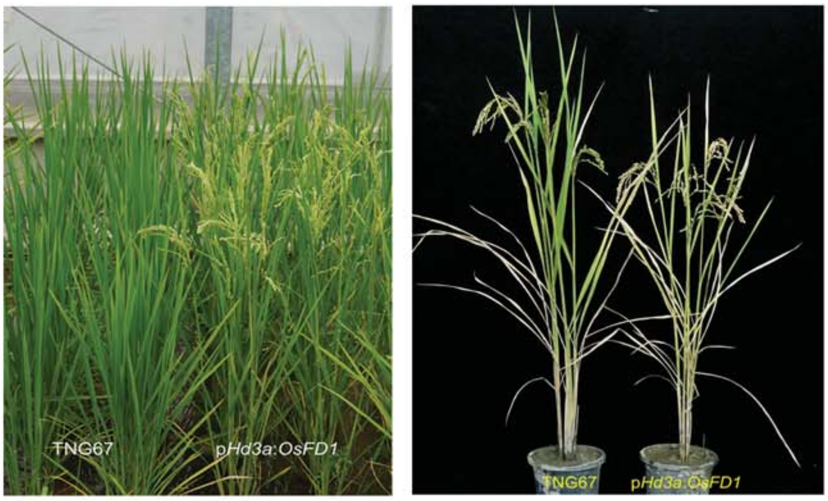

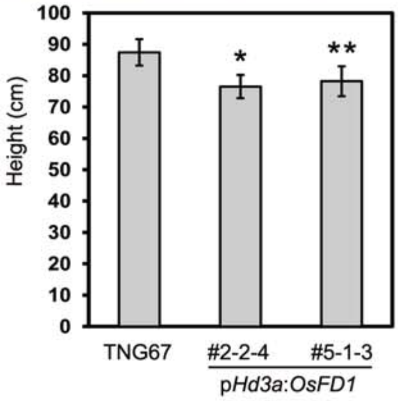

Genotype	Heading date
TNG67	101.6 ± 3.2
p*Hd3a*:*OsFD1* #2-2-4	85.7 ± 2.8
p*Hd3a*:*OsFD1* #5-1-3	88.5 ± 3.8
p*Hd3a*:*OsFD1* #8-3-3	86.1 ± 3.3

Genotype	Length	Width
TNG67	7.22 ± 0.20	3.61 ± 0.15
p*Hd3a*:*OsFD1* #2-2-4	7.28 ± 0.21	3.68 ± 0.17
p*Hd3a*:*OsFD1* #5-1-3	7.25 ± 0.24	3.60 ± 0.18

B

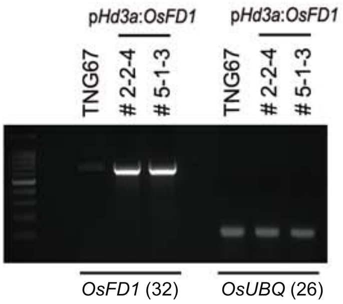

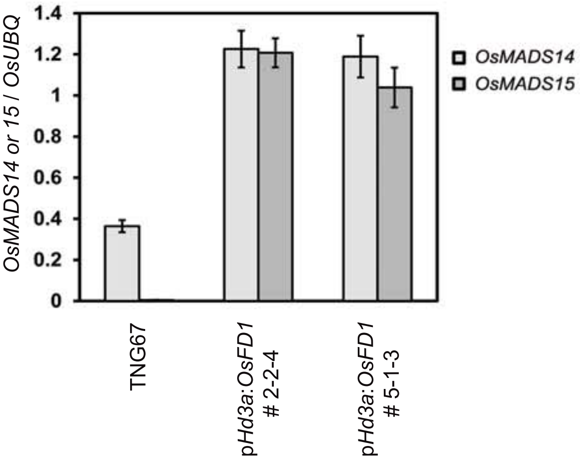

Supplementary figure 7

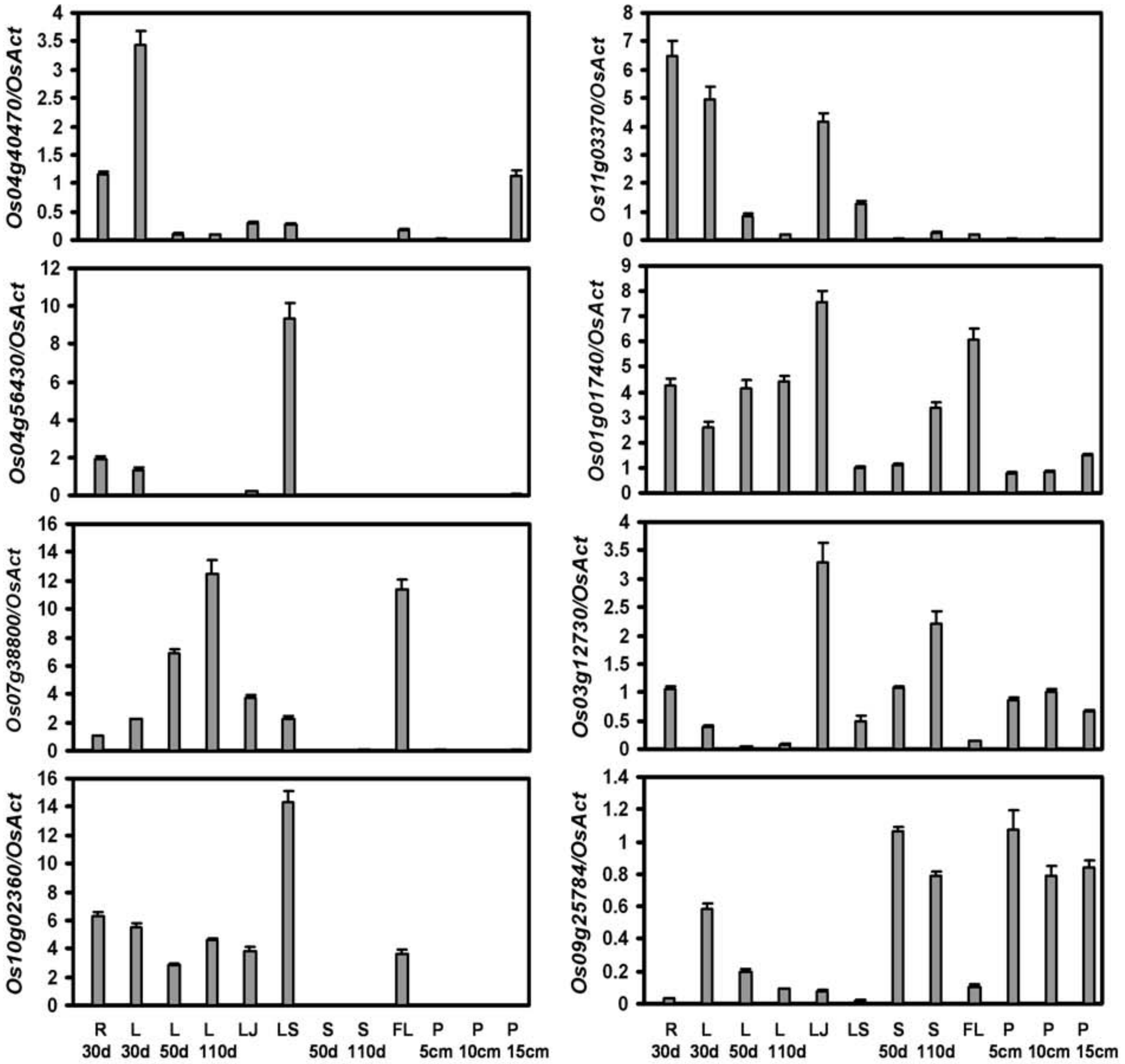

Supplementary figure 8

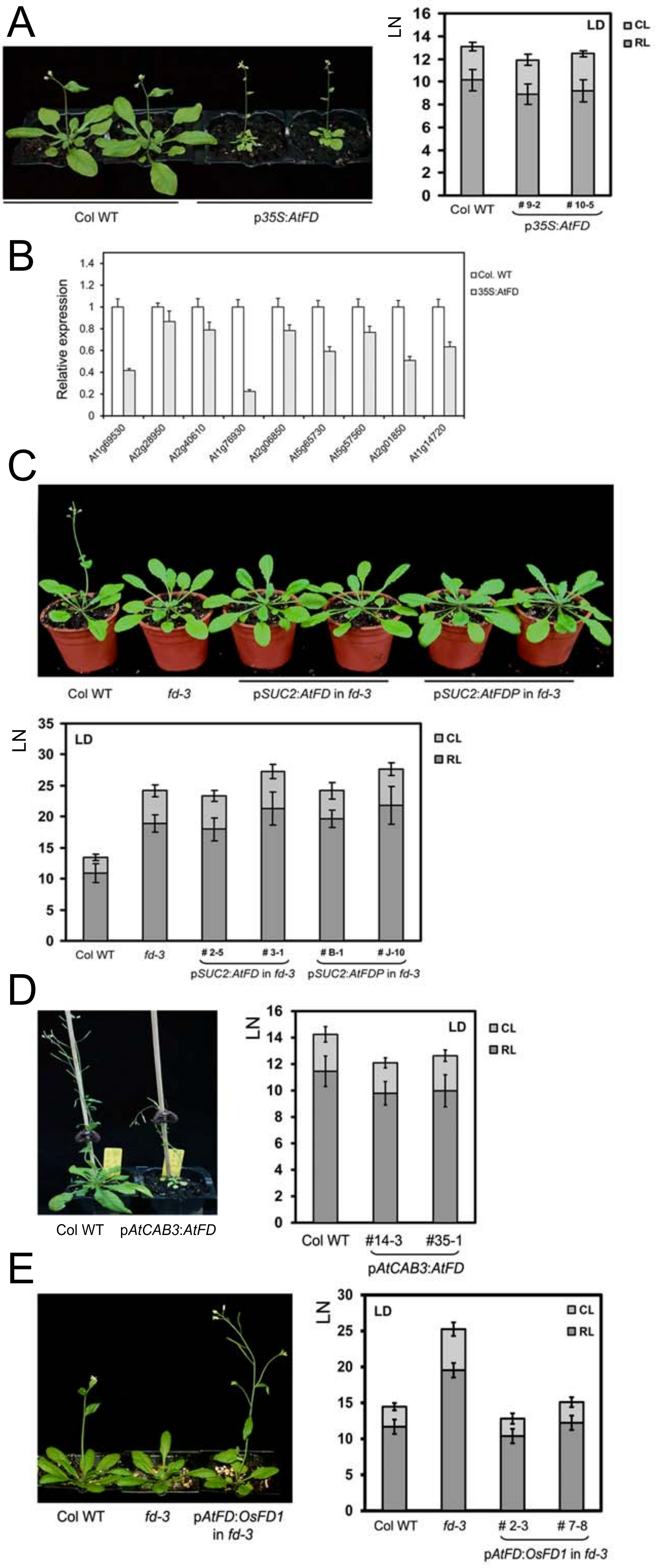

Supplementary figure 9

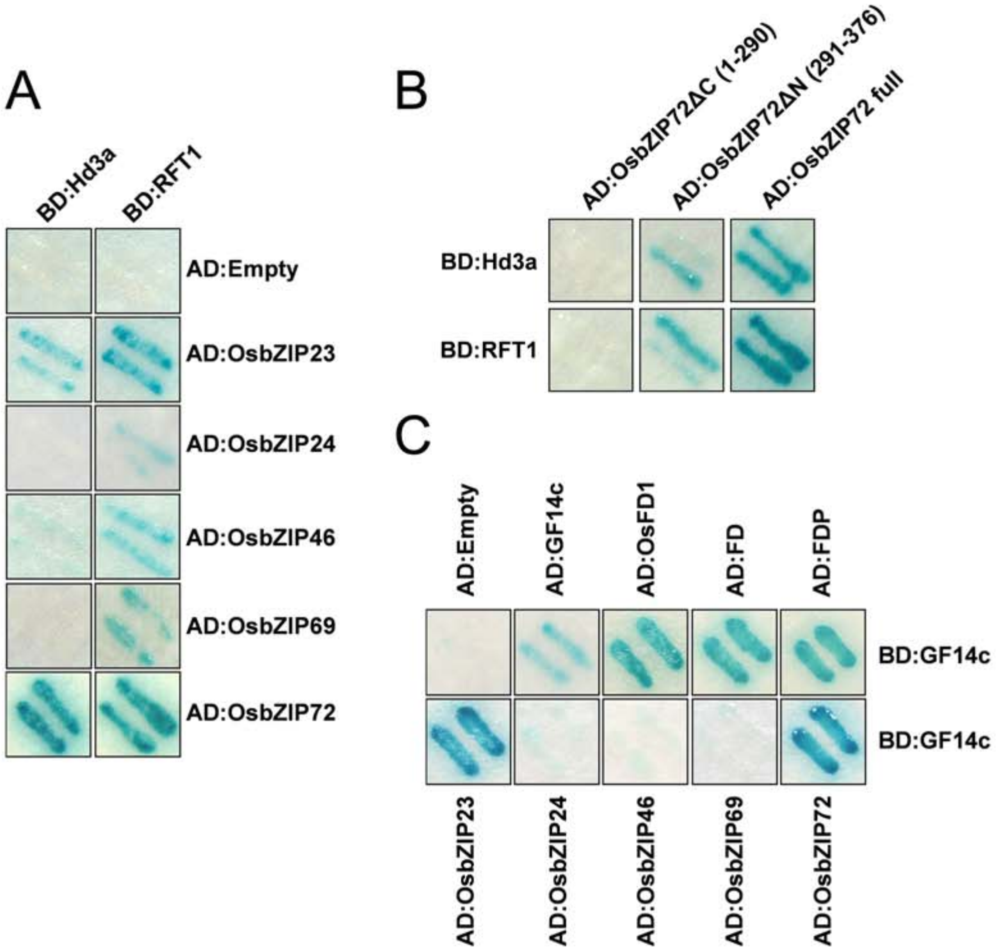

Supplementary figure 10

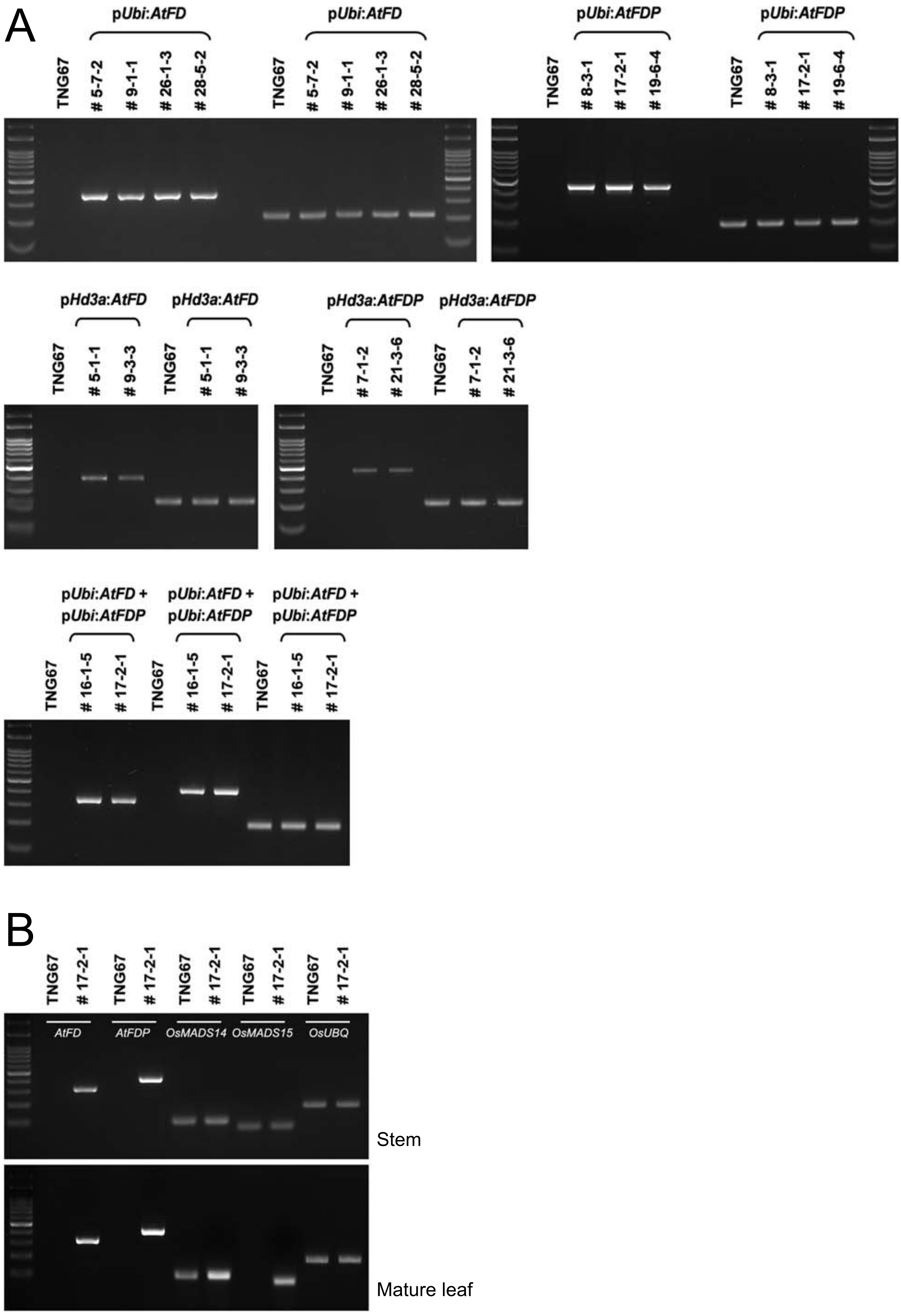

Supplementary figure 11

Supplementary Table S1.

Gene	RT and qRT-PCR Primers
AtFD	5' CTAAACCGACACAGCCCTCATC 3'
	5' GCATGAGCGTTTGAGAGGTGATG 3'
AtFDP	5' AGATCAACAACCATAGTGC 3'
	5' GACAACCAAACTTTGCAGATT 3'
UBQ only for RT-PCR (Rice)	5' GGTCATCCCGAGCCTCTGTT 3'
	5' GCAAATGAGCAAATTGAGCA 3'
OsMADS14	5' CGGTTGCGAGACGAGGAA 3'
	5' GAAAGACGGTGCTGGACGAA 3'
OsMADS15	5' CGTCGTCGGCCAAACAG 3'
	5' TGACTTCAATTCATTCAAGGTTGCT 3'
Os04g40470	5' CAACCTCCTCTTCTGCTTCG 3'
	5' GCACGAGAGGGTTCTTCTTG 3'
Os04g56430	5' CCTCCCAGGAGATCCTCTTC 3'
	5' GGGCGTAGCACTCGTTGTAG 3'
Os07g38800	5' AGTTCGATGTGGAGGAGGTG 3'
	5' GTAGCTCGGCGATAGGTCAG 3'
Os10g02360	5' GGATCACGAACACCTTGTCTCGC 3'
	5' CAGTGAAGCTCCATCGGCTGTG 3'
Os11g03370	5' CCTTCGTCTCAACCTCAAGGATGAG 3'
	5' GTCGAGCTGGAACAAGTCGTCG 3'
Os01g01740	5' GCTGTTGCTGTTGCCTTGAAGAATGC 3'
	5' CTGGTGATGGTGATGGTGCAGC 3'
Os03g12730	5' GGCTACATTGCTCCCGAGTACG 3'
	5' CGTAGAAGACGTGCGTCAGCTC 3'
Os09g25784	5' GAGCTGCACATTGGAAGCTTGATG 3'
	5' GTCTGCCAGCAGGAGAGGCTC 3'
Os03g43650 (Extensin)	5' ATCCATCCATGCATCCAAAT 3'
	5' CCACACCAAACACACACACA 3'
Os05g08770 (Extensin)	5' TGCATTGGTTGGTGTCTGTT 3'
	5' CTCCTTGCAAAGCCAAACTC 3'
Os04g15840 (Expansin1)	5’ AGTGACGCTTCAGGAACGAT 3’
	5’ TAGTCGCACATGATCCGGTA 3’
Os01g60770 (Expansin2)	5' CTCCAGATCGGCGTGTACC 3'
	5' AGTAGGAGTGCCCGTTGATG 3'
Os05g19570 (Expansin3)	5’ TCACGGTCACAAGCTCAAAG 3’
	5’ TCCCCGTCAACTACAAGAGG 3’

Os04g51460 (OsXTH1)	5' GATCCGGGAGATGAAGAACCA 3'
	5' TCCATCTGCTGGTCGTACCC 3'
Os11g33270 (OsXTR1)	5' GGAGCCGTACATCCTGCAGA 3'
	5' AGGCTGGAGTAGAGCTTCATCG
Os06g48160 (OsXTR2)	5'-CCCAGTCCTTCCACACCTAC-3'
	5'-GCCTCGTGGTTCTTGAAGTC-3'
Os02g46910 (OsXTR3)	5' TCAAGTTGCCCAAGGCTTAC 3'
	5' TCGTGCGTCTTCTCGTACAC 3'
Os07g39480 (OsWRKY78)	5' GGAAACCCCAATCCACGGAGTTAC 3'
	5' GCTTCCTGACTGGGCATCCTGTAT 3'
Os02g32660 (OsSBE3)	5' CAGGCCCTACTCGTTCTCAG 3'
	5' AGTCTATCCGGCATTCATCG 3'
Os08g40930 (ISA1)	5’ TCTGACTTGCAACGGTTCTG 3’
	5’ TCTCGCCTTTCGTTTCATCT 3’
Actin only for qPCR (Rice)	5' CTCCCCCATGCTATCCTTCG 3'
	5' TGAATGAGTAACCACGCTCCG 3'
UBQ only for qPCR (Rice)	5' AACCAGCTGAGGCCCAAGA 3'
	5' ACGATTGATTTAACCAGTCCATGA 3'
At1g69530 (AtEXPA1)	5' TAACCCTCCTCAGCAGCAT 3'
	5' CTTCTCACGCACGGCACT 3'
At2g28950 (AtEXPA6)	5' TTCTGGAACAA TGGGAGGAG 3'
	5' AGCAAAGTT CGGAGGACAGA 3'
At2g40610 (AtEXPA8)	5' CGCCGTCTCAATCAAAGGCTCAAA 3'
	5' ACCACCTTGGTAGGTTTGTCCGAA 3'
At1g76930 (AtEXT4)	5' CAAATCACCACCTCCTCCTC 3'
	5' TCAATTCTCCCGTCAACGAT 3'
At2g06850 (AtXTH4)	5' GGCTTATCACACTTACTCAATCCTTTG 3'
	5' TTCGGATTGGTATGTTGTCAACA 3'
At5g65730 (AtXTH6)	5' CGGTCACCGCTTTCTACATGA 3'
	5' CAAGAACTCAAAATCTAGCTCGTCTCT 3'
At5g57560 (AtXTH22)	5' GAAACTCCGCAGGAACAGTC 3'
	5' TGTCTCCTTTGCCTTGTGTG 3'
At2g01850 (AtXTH27)	5' CGGTTAACGCTCGATGAAAGA 3'
	5' GCACTGAAGAATCCATGCAAGTA 3'
At1g14720 (AtXTH28)	5' AGTATCCTTTGGTCTCTATCTCACATCA 3'
	5' GCCGTACGTTTGACTTCTCTGA 3'
AtPEX4 (At5g25760)	5' TTACGAAGGCGGTGTTTTTC 3'
	5' GGCGAGGCGTGTATACATTT 3'

Arabidopsis Act	5’ GGTAACATTGTGCTCAGTGGTGG 3’
	5’ AACGACCTTAATCTTCATGCTGC 3’
